# The incorporation loci of H3.3K36M determine its preferential prevalence in chondroblastomas

**DOI:** 10.1038/s41419-021-03597-9

**Published:** 2021-03-24

**Authors:** Yanjun Zhang, Dong Fang

**Affiliations:** Zhejiang Provincial Key Laboratory for Cancer Molecular Cell Biology, Life Sciences Institute, Zhejiang University, 310058 Hangzhou, Zhejiang China

**Keywords:** Mechanisms of disease, Histone post-translational modifications

## Abstract

The histone H3.3K36M mutation, identified in over 90% of chondroblastoma cases, reprograms the H3K36 methylation landscape and gene expression to promote tumorigenesis. However, it’s still unclear how the H3K36M mutation preferentially occurs in the histone H3 variant H3.3 in chondroblastomas. Here, we report that H3.3K36M-, but not H3.1K36M-, mutant cells showed increased colony formation ability and differentiation defects. H3K36 methylations and enhancers were reprogrammed to different status in H3.3K36M- and H3.1K36M-mutant cells. The reprogramming of H3K36 methylation and enhancers was depended on the specific loci at which H3.3K36M and H3.1K36M were incorporated. Moreover, targeting H3K36M-mutant proteins to the chromatin inhibited the H3K36 methylation locally. Taken together, these results highlight the roles of the chromatic localization of H3.3K36M-mutant protein in the reprogramming of the epigenome and the subsequent induction of tumorigenesis, and shed light on the molecular mechanisms by which the H3K36M mutation mainly occurs in histone H3.3 in chondroblastomas.

## Introduction

The epigenetic landscape is often altered and dysregulated to drive carcinogenesis. Recently, heterozygous mutations in histone H3 have been identified in pediatric bone tumors^1^. For example, >95% of chondroblastoma cases carry a somatic mutation in the *H3F3B* gene (which encodes the histone H3 variant H3.3) that substitutes lysine 36 (K36) with a methionine (H3.3K36M)^1^. Currently, additional histone mutations are being identified and characterized in tumors^2^.

H3K36 can be mono-, di-, and tri-methylated (H3K36me1/H3K36me2/H3K36me3) to regulate RNA splicing^3^, DNA methylation^4,5^, DNA repair^6^, gene transcription^7^, imprinting^8^, as well as m^6^A RNA modification^9^. In the human genome, there are 13 genes encoding canonical histone H3 (H3.1/H3.2), which are assembled into nucleosomes during S phase of the cell cycle^10^. Two genes encode histone H3 variant H3.3, which is assembled into nucleosomes in a replication-independent manner^11^. In chondroblastoma cases, the H3K36M mutation is mainly detected in the *H3F3B* gene, which encodes one copy of H3.3^1,12^. We and others have found that H3.3K36M-mutant protein inhibits the enzymatic activities of at least two H3K36 methyltransferases, MMSET and SETD2, which results in a global reduction of H3K36 methylation^13–16^. In addition to the global reduction in H3K36 methylation, the antagonizable recruitment of the Polycomb complex increases in the intergenic region, leading to elevated H3K27me3 levels^13,14,17^. More importantly, cells carrying the H3.3K36M mutation exhibited cancer phenotypes, especially differentiation defects.

However, both the H3.1K36M and H3.3K36M mutations inhibited H3K36 methylation on wild-type histone H3^14,18^. The inhibition effects of H3K36M-mutant protein on H3K36 methylation might be a general consequence of the suppression of histone methyltransferases without locus-specific effect. Since the H3.3K36M and H3.1K36M might be incorporated at different regions of chromatin as wild-type H3.3 and H3.1, we speculated that the incorporation loci of H3.3K36M and H3.1K36M determined the preferential prevalence of H3.3K36M in chondroblastomas. This may further explain how the H3.3K36M-mutant protein reprogrammed the epigenome. In addition to using previously constructed H3.3K36M cells, we further knocked-in a heterozygous K36M mutation in one H3.1 gene, *HIST1H3D*. Only H3.3K36M-mutant cells showed increased colony formation and differentiation defects. The epigenomes were reprogramed according to locus-specific incorporation of mutant proteins. Targeting of truncated H3K36M-mutant protein to chromatin inhibited the H3K36 methylation locally. Together, our results show that the H3.3K36M mutation reprograms the epigenome and gene expression landscape depending on the genomic loci of its incorporation, thus leading to an enrichment of H3.3K36M mutation in chondroblastomas.

## Results

### The H3.1K36M mutation has no effect on colony formation or differentiation

**Fig. 1:**
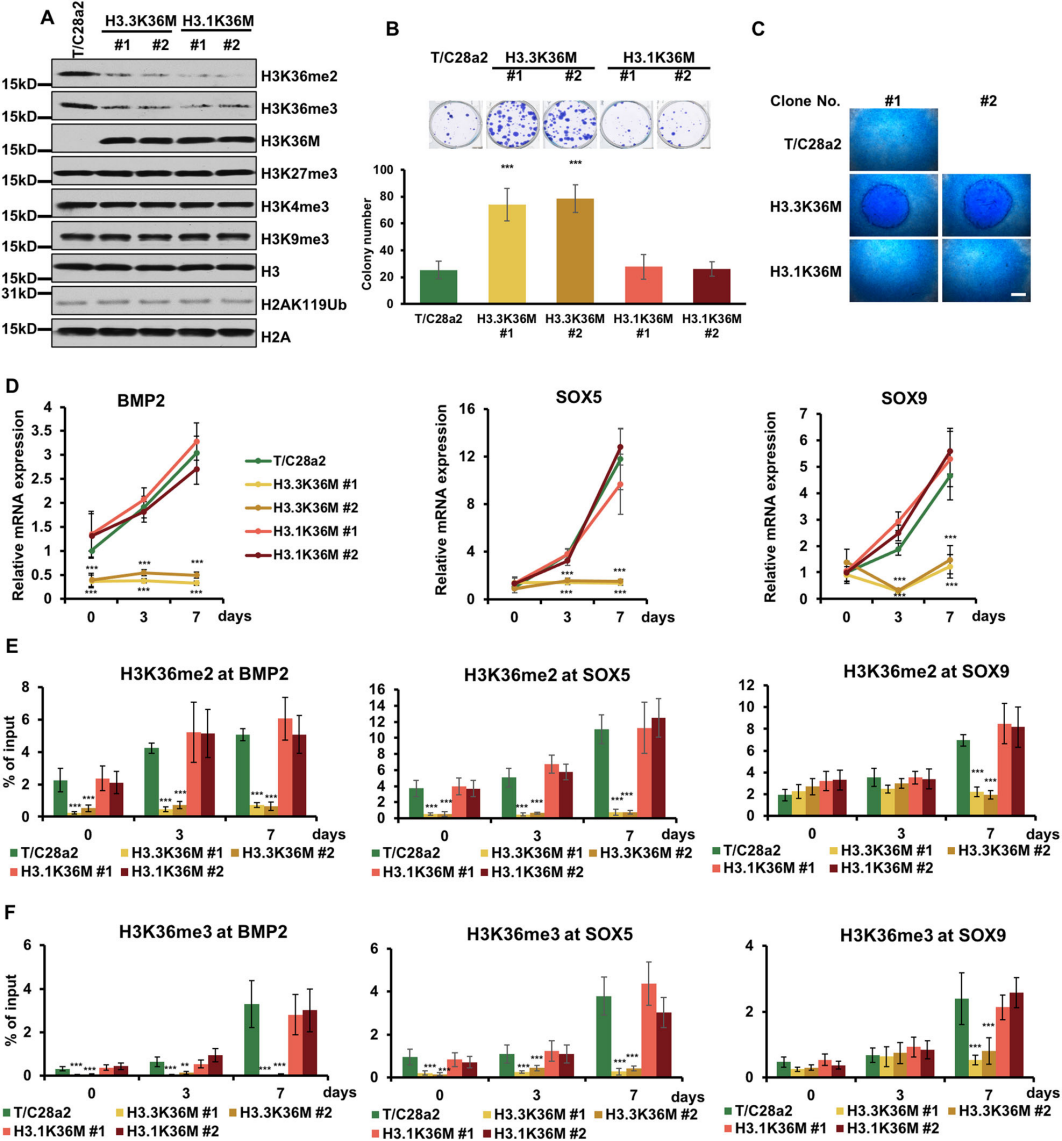
H3.3K36M-mutant cells show cancer-related cellular phenotypes. **A** H3.3K36M and H3.1K36M knock-in reduced the H3K36me2 and H3K36me3 levels in chondrocyte cell lines. Cell extracts were analyzed via western blotting using the indicated antibodies. **B** H3.3K36M-mutant cells showed increased colony formation ability. The results are represented by the mean ± SD (*N* = 3 independent replicates, ***p < 0.001). **C** H3.1K36M-mutant cells undergo normal chondrogenic differentiation. Alcian blue staining of differentiated micromasses are shown. Scale bars: 50 μm. **D** The expression levels of chondrogenic differentiation markers, including BMP2, SOX5, and SOX9, were analyzed via RT-RCR and normalized to GAPDH. The expression levels of the genes in T/C28a2 cells before differentiation were normalized as 1. The samples of day 0 were collected after seeding as micromass. **E** The H3K36me2 enrichment at BMP2, SOX5, and SOX9 was analyzed. WT, H3.3K36M-, and H3.1K36M-mutant cells were differentiated for 0, 3, and 7 days. At each time point, ChIP DNA at the indicated loci were calculated against the input DNA. **F** As in **E**, except the H3K36me3 enrichment at the indicated loci was analyzed. The results in **D**–**F** are represented by the mean ± SD (*N* = 3 independent replicates, **p < 0.01, ***p < 0.001).

To uncover how H3.3K36M mutation specifically induces tumorigenesis in chondroblastoma, we designed to knock-in the H3.1K36M mutation into immortalized human chondrocyte cells, T/C28a2. We used CRISPOR^19^ and found a sgRNA with the highest score in the *HIST1H3D* gene (Supplementary Fig. S1A). Two independent clones with a heterozygotic K36M mutation were then generated by CRISPR/Cas9 system (Supplementary Fig. S1B). The potential three off-target sites were confirmed with no mutations (Supplementary Fig. S1C). Like H3.3K36M mutation, the H3.1K36M mutation reduced the levels of H3K36me2/me3, while other analyzed histone marks showed no obvious changes (Fig. 1A).

Given our previous observation that H3.3K36M-mutant cells exhibit several hallmarks of cancer phenotypes^13^, we next examined whether the H3.1K36M mutation could induce the cancer-related phenotypes. Surprisingly, while the H3.3K36M mutation increased the colony formation, as previous discovered^13^, the H3.1K36M mutation did not (Fig. 1B). More importantly, H3.1K36M cells underwent chondrogenic differentiation as the parental cells, whereas H3.3K36M cells formed denser micromasses (Fig. 1C). Consistent with the micromass formation, the expression levels of chondrogenic differentiation markers, including BMP2, SOX5, and SOX9, were reduced only in H3.3K36M-mutant cells (Fig. 1D and Supplementary Fig. S1D). Moreover, the H3K36 methylations at BMP2, SOX5, and SOX9 remained unchanged in H3.3K36M-mutant cells, but increased in wild-type and H3.1K36M-mutant cells during differentiation (Fig. 1E, F). Overexpression of BMP2 or SOX5 alone in H3.3K36M-mutant cells could not rescue the differentiation defect, suggesting that several differentiation markers were collectively reduced by H3.3K36M-mutant proteins to induce the differentiation defect (Supplementary Fig. S1E). Compared with the parental cells, mutant cells had no apparent defects in mismatch repair, chromatin accessibility, or cellular proliferation (Supplementary Fig. S1F–I). Furthermore, both mutant cells were more resistant to staurosporine-induced apoptosis (Supplementary Fig. S1J). H3K36M-mutant protein behaved like an inhibitor of H3K36 methyltransferases, however, overexpressed wild-type H3 didn’t rescue the reduction of H3K36 methylation (Supplementary Fig. S1K). We speculated that only a little amount of mutant proteins could inhibit the H3K36 methylation. Overexpressing of H3.3K36M and H3.1K36M increased the H3K27me3 levels (Supplementary Fig. S1L) that was not observed in the knock-in cells. Although the ectopic H3K36M-mutant proteins were expressed at low levels compared with endogenous H3, it’s more than that in the knock-in system (Supplementary Fig. S1M). In line with this, H3K36me2/me3 decreased more in the overexpression cells. It is possible that the increase of H3K27me3 was at a moderate level, which was not detected by western blot. To further evaluated whether the cancer-related phenotypes in H3K36M cells were the direct effects of changed H3K36 methylation, we overexpressed H3.3K36R and H3.1K36R, respectively (Supplementary Fig. S1N). The growths were similar in all cell lines (Supplementary Fig. S1O). Only H3K36M overexpressed cells showed increased colony formation and were resistant to staurosporine-induced apoptosis (Supplementary Fig. S1P, Q). Taken together, these data imply that only H3.3K36M-mutant cells exhibit strong cancer-related hallmarks, which might be caused by distinct effects of H3.3K36M and H3.1K36M.

### The H3.3K36M and H3.1K36M mutations alter different subsets of genes

**Fig. 2:**
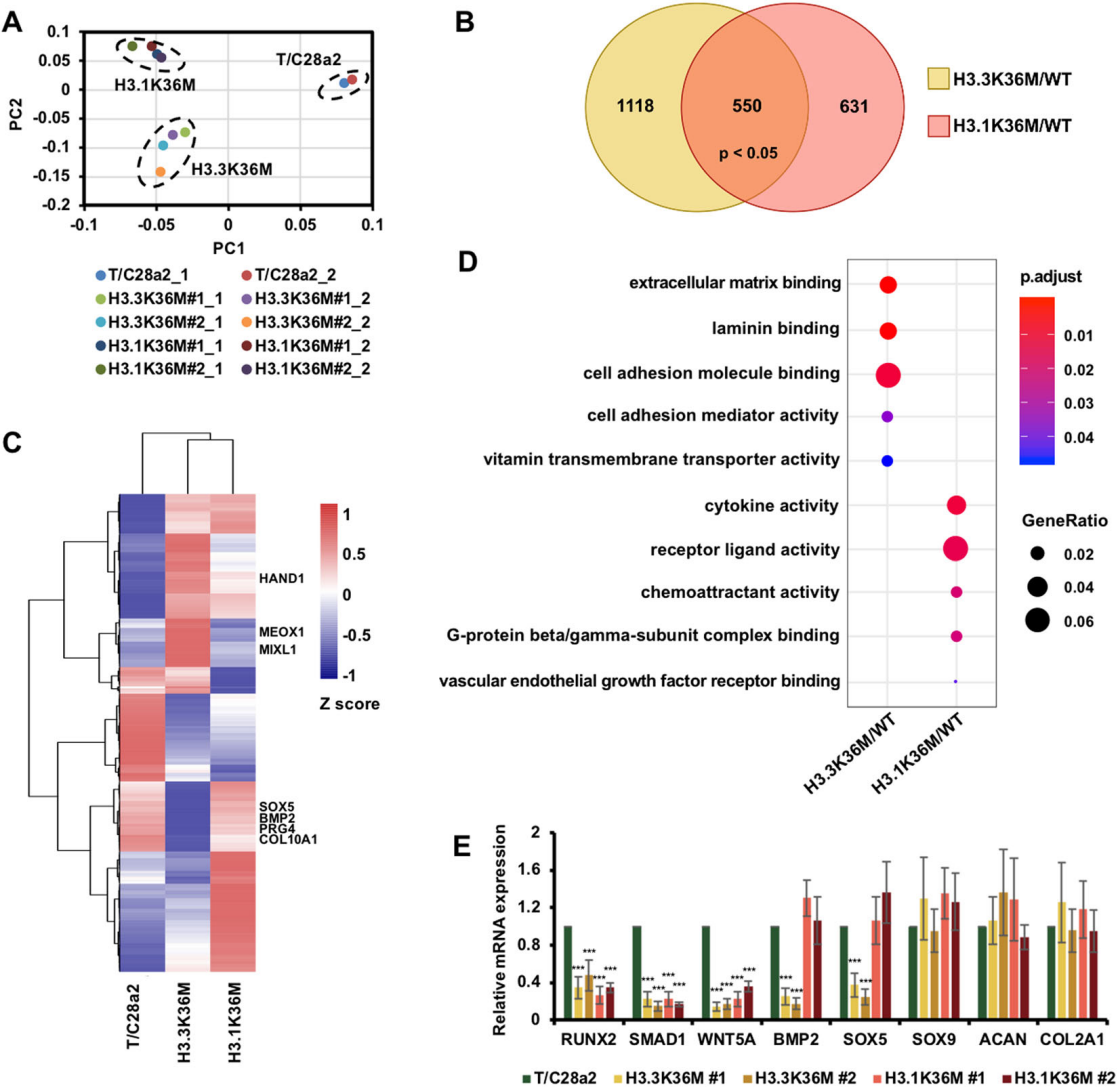
H3.3K36M and H3.1K36M alter the expression levels of different sets of genes. **A** The principal component analysis (PCA) plot of the transcriptomic data from wild-type (WT), H3.3K36M, and H3.1K36M cells. The transcriptomic data from the independent clones of H3.3K36M- and H3.1K36M-mutant cells and two replicates were presented. **B** Venn diagram illustrating the overlap between the genes altered in the H3.3K36M- and H3.1K36M-mutant cells compared with their levels in WT cells. The RNA-seq data from two independent clones were merged to analyze differentially expressed genes. **C** Gene expression heatmap for wild-type, H3.3K36M, and H3.1K36M cells. All the changed genes between wild-type and H3K36M-mutant cells were collected for this clustering analysis. To represent the consistently changes of RNA-seq data, we firstly merged two replicates of each cell line and then merged the RNA-seq data of two independent clones of H3.3K36M and H3.1K36M. **D** The scatterplot shows the gene ontology (GO) analysis of the differentially expressed genes. The colors indicate the *p* value. The size was scaled according to the fractions of genes in the GO terms. **E** The expression levels of chondrogenic differentiation-related marker genes were analyzed via RT-PCR. The data are represented by the mean ± SD (*N* = 3 independent replicates, ***p < 0.001).

To understand how the H3.3K36M and H3.1K36M mutations lead to the specific cellular phenotypes, we compared the gene expression profiles via RNA-seq. Two replicates of each cell line were sequenced and showed good correlation (Fig. 2A). 1668 and 1181 genes were altered in H3.3K36M- and H3.1K36M-mutant cells, respectively. Only 550 genes were both altered in mutant cells, of which 302 genes and 248 genes were downregulated and upregulated in mutant cell lines, respectively (Fig. 2B). Gene ontology analysis showed that these 550 changed genes in both mutants were enriched in binding and receptor ligand activity (Supplementary Fig. S2A). These genes were enriched in binding and receptor ligand activity, suggesting that these altered genes were involved in the regulation of cell signaling pathways. Moreover, gene set enrichment analysis revealed that these commonly altered genes were significantly enriched in the regulation of apoptotic pathway (Supplementary Fig. S2B), which was consistent with the finding that mutant cells were resistant to staurosporine-induced apoptosis (Supplementary Fig. S1G). In addition, the altered genes in the H3.3K36M- and H3.1K36M-mutant cells were enriched in different groups when clustered with wild-type cells (Fig. 2C and Supplementary Fig. S2C). The altered genes were mainly assigned to the extracellular matrix binding and cell adhesion functions, which may alter colony formation and differentiation in H3.3K36M-mutant cells (Fig. 2D). In addition, the altered genes were associated with cytokine activity and receptor ligand activity, which may change the response of cell signaling in H3.1K36M-mutant cells.

Furthermore, we confirmed the expression levels of chondrogenic differentiation marker genes via reverse transcription-PCR (RT-PCR; Fig. 2E). Compared with wild-type cells, the levels of *BMP2* and *SOX5*, decreased in H3.3K36M-mutant cells but remained similar in H3.1K36M-mutant cells. The levels of *RUNX2*, *SMAD1*, and *WNT5a*, which are early differentiation markers, decreased in all of the mutant cells. The levels of other tested markers were not changed. Together, these data suggest that the H3.3K36M mutation alters the expression levels of differentiation-related genes that may, in turn, induce the cancer-related phenotypes.

### H3K36 methylations are differentially reprogrammed in H3.3K36M- and H3.1K36M-mutant cells

**Fig. 3:**
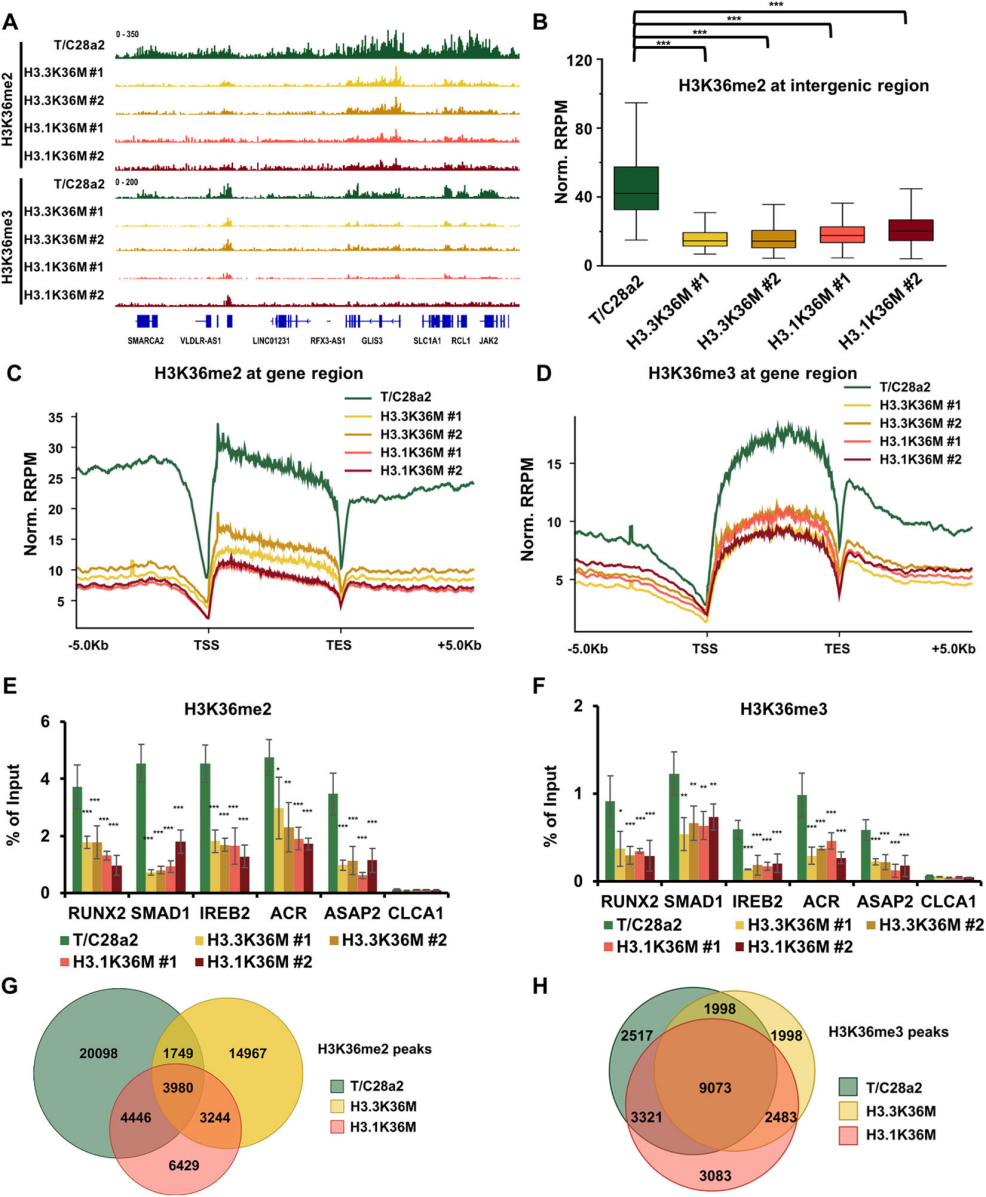
H3K36 methylation is differentially reprogrammed in H3.3K36M- and H3.1K36M-mutant cells. **A** Integrative genomics viewer (IGV) tracks representing the distributions of H3K36me2 and H3K36me3 in wild-type (WT), two H3.3K36M-, and two H3.1K36M-mutant cell lines. **B**, **C** The normalized read distribution profiles of H3K36me2 at intergenic regions (**B**) and gene bodies (**C**) in different cell lines. TSS transcription start sites, TES transcription end sites, Norm. RRPM normalized reference-adjusted reads per million. ***p < 0.001. **D** The normalized read distribution profiles of H3K36me3 at gene bodies in different cell lines. **E**, **F** H3K36me2 (**E**) and H3K36me3 (**F**) enrichment were analyzed via ChIP-qPCR in WT and H3K36M-mutant cell lines. The data are represented by the mean ± SD (*N* = 3 independent replicates, *p < 0.05, **p < 0.01, ***p < 0.001). **G**, **H** Venn diagram illustrating the overlap of H3K36me2 (**G**) and H3K36me3 (**H**) peaks in WT, H3.3K36M-, and H3.1K36M-mutant cells, respectively. The peaks from independent clones were pooled.

To more definitively compare the changes in H3K36me2 and H3K36me3 levels in mutant cells, we performed ChIP-Rx. Individual browser track views revealed that H3K36me2 was enriched at gene bodies, as well as intergenic regions and that H3K36me3 was enriched at gene bodies (Fig. 3A and Supplementary Fig. S3A). Furthermore, the normalized H3K36me2 ChIP-seq read densities at intergenic regions, and gene bodies dramatically decreased in H3.3K36M- and H3.1K36M-mutant cells (Fig. 3B, C). Likewise, the H3K36me3 level was reduced throughout the gene bodies in the mutant cell lines (Fig. 3D). The reductions of H3K36me2 and H3K36me3 at five genes were confirmed via ChIP-qPCR (Fig. 3E, F).

To further analyze the H3K36 methylation distribution profile, we called H3K36me2 and H3K36me3 ChIP-seq peaks by combining two independent clones. The reductions in H3K36me2 and H3K36me3 were further supported by heatmaps, representing the H3K36 methylation level at peak regions (Supplementary Fig. S3B, C). We identified 30,273, 23,940, and 18,099 H3K36me2 peaks in wild-type, H3.3K36M-, and H3.1K36M-mutant cells, respectively. Over 70% and 50% of the peaks in H3.3K36M and H3.1K36M cells were unique in the mutant cells, respectively (Fig. 3G). Moreover, the correlations of H3K36me2 ChIP-seq data were low among wild-type, H3.3K36M-, and H3.1K36M-mutant cells (Supplementary Fig. S3D), suggesting that H3K36me2 is reprogrammed to different degrees in H3.3K36M- and H3.1K36M-mutant cells. The total numbers of H3K36me3 peaks and correlations were similar among the wild-type and mutant cells (Fig. 3H and Supplementary Fig. S3E). Like we previously observed in H3.3K36M cells^13^, the gene expression levels and the enrichments of H3K36me2/H3K36me3 in the H3K36 methylation reduced gene bodies were positively correlation in H3.1K36M cells (Supplementary Fig. S3F, G). Together, these results indicate that H3K36 methylation is differentially reprogrammed in mutant cells to alter their gene expression profiles.

### The H3.3K36M- and H3.1K36M-mutant proteins inactivate different enhancers associated with their genomic loci

As previously detected in H3.3K36M-mutant cells^14,20^, H3K327me3 significantly increased in the intergenic regions, but not at the promoter regions in H3.1K36M-mutant cells (Supplementary Fig. S4A, B). Over 50% of the H3K27me3 peaks were unique in H3.3K36M-mutant cells. By contrast, <5% of the H3K27me3 peaks were unique in H3.1K36M-mutant cells (Supplementary Fig. S4C). The H3K27me3 across the whole genome was highly correlated between the two independent clones, indicating that the ChIP-seq results were repeatable and stable (Supplementary Fig. S4D). Together, these data suggest that the H3.3K36M mutation reprograms H3K27me3 to new genomic loci on the chromatin, which likely leads to a unique epigenetic landscape in the intergenic regions.

**Fig. 4:**
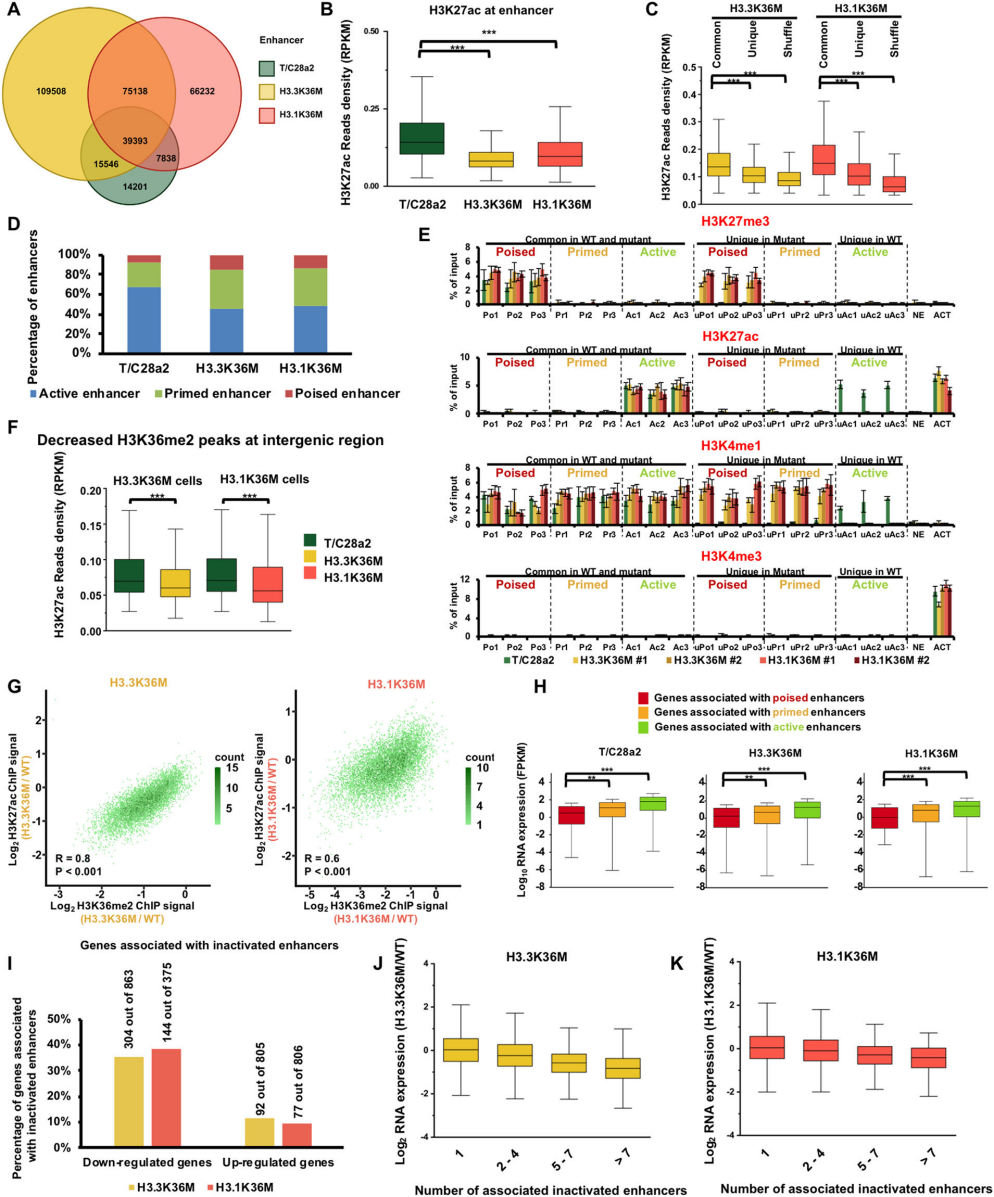
The H3.3K36M and H3.1K36M mutations inactivate enhancers. **A** Venn diagram illustrating the overlap of enhancers in wild-type, H3.3K36M-, and H3.1K36M-mutant cells. Enhancers were defined as genomic loci with H3K4me1 peaks, but not overlapped with H3K4me3 peaks or annotated promoters. The enhancers from independent H3.3K36M- and H3.1K36M-mutant clones were pooled. **B** The normalized H3K27ac read density at enhancers in different cell lines. ***p < 0.001. **C** The normalized H3K27ac read density at common and unique enhancers in H3.3K36M- and H3.1K36M-mutant cells. A set of shuffled peaks corresponding to unique peaks was used to analyze the enrichment of the ChIP-seq reads. ***p < 0.001. **D** Percentages of classified enhancers. Enhancers were classified according to their enrichment in H3K27ac and H3K27me3. H3K4me1+/H3K27ac+ loci correspond to active enhancers; H3K4me1+/H3K27me3+ loci correspond to primed enhancers, and H3K4me1+/H3K27ac− loci correspond to poised enhancers. **E** The H3K27me3, H3K27ac, H3K4me1, and H3K4me3 enrichments at the classified enhancers were analyzed via ChIP-qPCR. The data are represented by the mean ± SD (*N* = 3 independent replicates). Po1, Po2, and Po3, poised enhancers in all cell lines. Pr1, Pr2, and Pr3, primed enhancers in all cell lines. Ac1, Ac2, and Ac3, active enhancers in all cell lines. uPo1, Upo2, and uPo3, unique poised enhancers in mutant cells, uPr1, uPr2, and uPr3, unique primed enhancers in mutant cells. uAc1, uAc2, and uAc3, unique active enhancers in wild-type cells. NE, intergenic locus without H3K27me3, H3K27ac, H3K4me1, and H3K4me3 was used as negative controls. ACT, genomic locus of *ACTB* which was actively expressed was used as the positive control for H3K27ac and H3K4me3. **F** H3K27ac decreased in the intergenic regions with decreased H3K36me2. The H3K27ac enrichment at H3K36me2-decreased intergenic regions was analyzed in H3.3K36M- and H3.1K36M-mutant cells. ***p < 0.001. **G** Correlations between the changes of H3K27ac and H3K36me2 in wild-type and mutant cells. The active enhancers in wild-type cells that were within the H3K36me2 peaks were chosen for the analysis. The correlations were assessed by Pearson product moment correlation. **H** Box plots showing the expression levels of genes associated with distinct classes of enhancers. FPKM transcript per million mapped reads. **p < 0.01, ***p < 0.001. **I** The percentage of upregulated and downregulated genes that were associated with inactivated enhancers. Genes that were significantly changed in H3.3K36M- and H3.1K36M-mutant cells were used for this analysis. **J**, **K** The expression changes of genes associated with different number of inactivated enhancers in H3.3K36M- (**J**) and H3.1K36M- (**K**) mutant cells.

Because H3K27me3 in intergenic regions is correlated to the activation of enhancers, we next focused on alterations at enhancers in wild-type and mutant cells. We next focused on enhancers by additional profiling H3K27ac, H3K4me1, and H3K4me3 (Supplementary Fig. S5A). Thus, we identified enhancers as genomic loci with H3K4me1 peaks, but not overlapped with H3K4me3 peaks or annotated promoters, which were defined as sequences 2 Kb upstream and 500 bp downstream of transcription start sites (TSSs) based on the NCBI RefSeq database^21^. We called 76,978 enhancers in wild-type cells, 239,585 enhancers in H3.3K36M-mutant cells, and 188,601 enhancers in H3.1K36M-mutant cells (Fig. 4A). Because the number of enhancers was dramatically increased in mutant cells, we analyzed whether the enrichments of H3K4me1 were changed in mutant cells. H3K4me1 ChIP-seq signal at intergenic region and gene bodies was not changed (Supplementary Fig. S5B, C), and the total levels of H3K4me1 were not notable altered (Supplementary Fig. S5D). The average distances to the closest TSS were ~40 and 68 kb in common and unique enhancers in H3.3K36M cells, respectively. And the average distances were ~36 kb in both common and unique enhancers in H3.1K36M cells (Supplementary Fig. S5E). Moreover, we found that the length of enhancers decreased in both the mutant cells (Supplementary Fig. S5F). Together, these results suggest that the increased number of enhancers in H3.3K36M- and H3.1K36M-mutant cells is due to the decreased length of enhancer, but not the overall enrichment of H3K4me1 on the chromatin.

To elucidate the activities of enhancers, we then compared the levels of active enhancer mark, H3K27ac, in cells. To our surprise, although the total numbers of enhancers increased in H3.1K36M-mutant cells, the H3K27ac level decreased at enhancers (Fig. 4B). The enrichments of H3K27ac and H3K4me1 at enhancers in all cell lines were confirmed via ChIP-qPCR (Supplementary Fig. S5G, H). To further characterize the H3K27ac in the mutant cells, we separated enhancers into two classes: unique enhancers in mutant cells, and common enhancers in both wild-type and mutant cells. The enrichment of the H3K27ac was lower at the unique enhancers than common enhancers in the mutant cells, indicating that the newly initiated enhancers were inactive (Fig. 4C). Because H3K27me3 and H3K27ac modifications were on the same H3K27 residue and may be modified as a Yin-Yang process, it’s possible that the decrease of H3K27ac was directly linked to the H3K27me3 increase. We compared the changes of H3K27ac and H3K27me3 at the H3K27me3 increased peaks in H3.3K36M and H3,1K36M-mutant cells, respectively. Around half of these H3K27me3 increased peaks showed a decrease and increase of H3K27ac, respectively (Supplementary Fig. S5I, J). Moreover, the correlation was low between the changes of H3K27ac and H3K27me3. It’s unlikely that the decrease of H3K27ac is caused by the increase of H3K27me3. To further profile the activation state of the enhancers, we classified enhancers as previously described^21^: enhancers with H3K27ac as active enhancers, enhancers with H3K27me3 as poised enhancers, and enhancers lacking H3K27me3 and H3K27ac as primed enhancers. The percentages of primed and poised enhancers increased in mutant cells (Fig. 4D). 32,249 (61.2%) and 29,256 (55.5%) of 52,672 active enhancers in wild-type cells were inactivated in H3.3K36M and H3.1K36M cells, respectively. The enrichments of H3K27me3, H3K27ac, H3K4me1, and H3K4me3 were further confirmed by ChIP-qPCR (Fig. 4E and Supplementary Fig. S5K). In mutant cells, the H3K27ac levels decreased in the intergenic H3K36me2 peaks with reduced H3K36me2 (Fig. 4F). The active enhancers, over 80% of which overlapped with H3K36me2 peaks in wild-type cells, were chosen to directly compare the effects of H3K36me2 changes on the H3K27ac. 8086 and 8117 of 8162 enhancers in wild-type cells exhibited decreased H3K36me2 in H3.3K36M and H3.1K36M cells, respectively. The changes of H3K27ac and H3K36me2 were positively correlated in the H3.3K36M and H3.1K36M cells (Fig. 4G). Moreover, H3K36M-mutant protein were enriched at the enhancers (Supplementary Fig. S5L). Together, these data suggest that the reduction of H3K27ac is directly associated with the decrease of H3K36me2.

To investigate the correlation between gene expression and enhancer activation, we assigned enhancers to the closest promoter within 500 kb as described^22^. Genes associated with more active enhancers showed higher expression levels (Supplementary Fig. S5M). In addition, genes associated with active enhancers showed the highest expression levels, followed by primed and poised enhancers (Fig. 4H). Genes with decreased transcripts were associated with the inactivated enhancers (35% in H3.3K36M cells, and 38% in H3.1K36M cells), whereas only 11% and 9% of genes with increased transcripts were assigned to the inactivated enhancers in H3.3K36M and H3.1K36M cells, respectively (Fig. 4I). Moreover, the expressions of genes associated with more inactivated enhancers decreased more in the mutant cells (Fig. 4J, K). We then used HOMER to annotate the motifs in the inactivated enhancers^23^. The Jun/AP1-binding motif was enriched at a lowest *p* value in mutant cells (Supplementary Fig. S5N). It may play as the master regulators for the changed genes. Interestingly, p53 was identified only in H3.3K36M cells, which was consistent with that p53 pathway were changed in tumor tissues^13^. Collectively, these data indicate that the enhancers are inactivated in H3.3K36M- and H3.1K36M-mutant cells, which may further reprogram gene expression.

### The H3K36 methylation reprogramming depends on the genomic loci of the H3K36M-mutant proteins

**Fig. 5:**
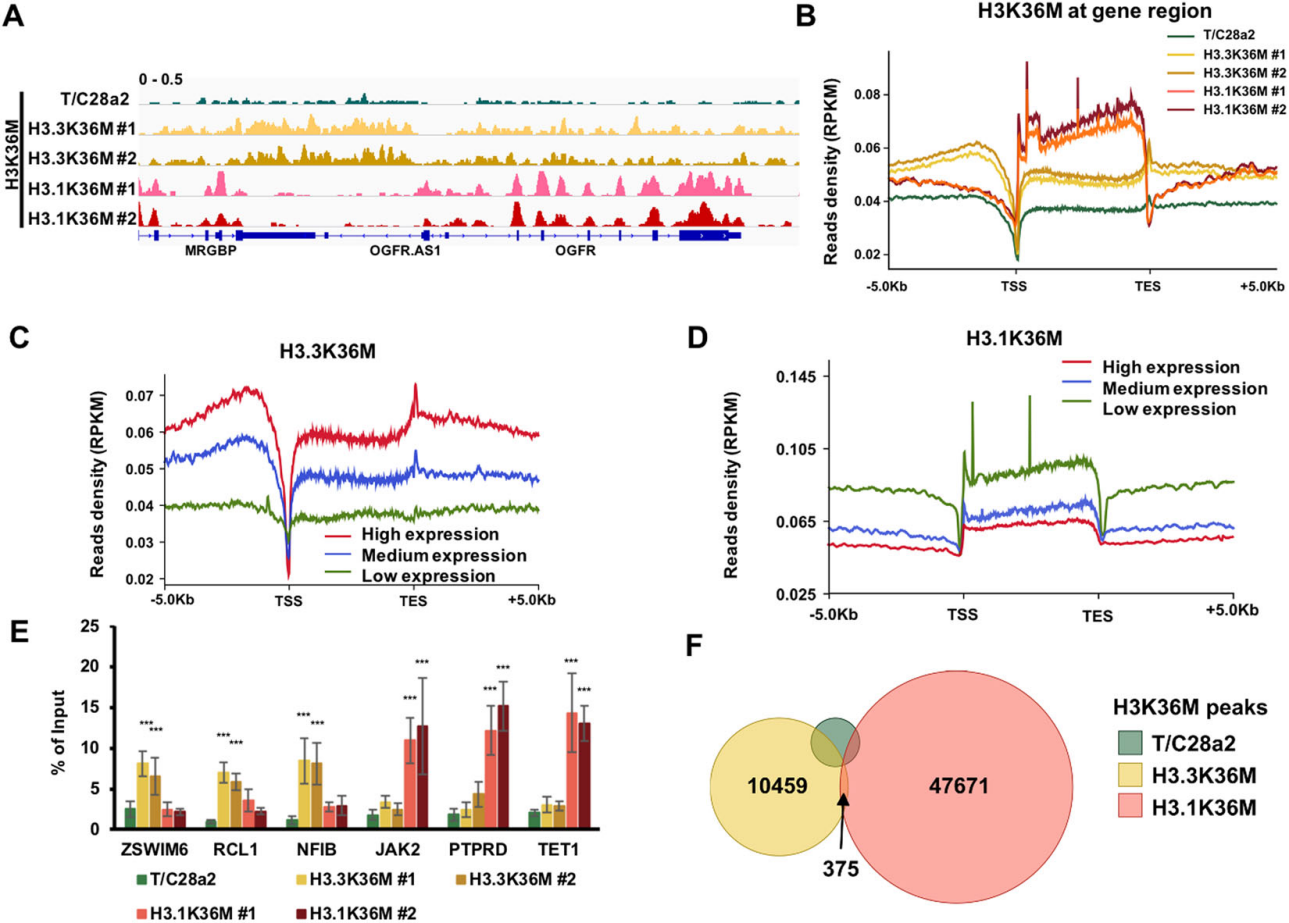
H3.3K36M is enriched in regions lacking H3.1K36M. **A** IGV tracks showing the chromatin distributions of H3K36M in wild-type (WT), H3.3K36M-, and H3.1K36M-mutant cell lines. **B** The distribution profiles of normalized H3K36M read density from 5 kb upstream of the TSS to 5 kb downstream of the TES. PRKM reads per kilobase million. **C**, **D** Normalized read density of H3K36M from 5 kb upstream of the TSS to 5 kb downstream of the TES in grouped genes with high, medium, and low expression levels. The average read densities of the H3K36M ChIP-seq in H3.3K36M- (**C**) and H3.1K36M- (**D**) mutant cells were calculated. The genes were separated into high expression, medium expression, and low expression groups based on their expression levels. **E** H3K36M enrichment analyzed via ChIP-qPCR. The data are represented by the mean ± SD (*N* = 3 independent replicates, ***p < 0.001). **F** Venn diagram illustrating the overlap of the H3K36M peaks in H3.3K36M- and H3.1K36M-mutant cells. The H3K36M peaks in independent H3.3K36M- and H3.1K36M-mutant clones were combined.

Since H3.3K36M and H3.1K36M both reprogrammed the epigenetic status and gene expression, we hypothesize that the enriched genomic loci of H3.3K36M and H3.1K36M were important for the induction of cancer-associated phenotypes in H3.3K36M-mutant cells. Individual browser track views (Fig. 5A) and distribution profiles around the gene regions (Fig. 5B) showed that the H3.3K36M and H3.1K36M proteins were exclusively enriched. Moreover, H3.3K36M was highly enriched in actively transcribed genes (Fig. 5C) and H3.1K36M was associated with repressed genes (Fig. 5D). The specific occupancies of H3K36M were further confirmed via ChIP-qPCR (Fig. 5E). By combining the ChIP-seq results from two independent clones, we identified 10,834 and 48,046 H3K36M peaks in H3.3K36M- and H3.1K36M-mutant cells, respectively (Fig. 5F). 3671 false-positive H3K36M peaks were recognized in wild-type cells. Moreover, H3K36M signal was exclusively detected at each peak in the corresponding cells (Supplementary Fig. S6A–C), and the correlations of H3K36M signal were low between cells with different genotypes (Supplementary Fig. S6D). Taken together, these data indicate that H3.3K36M and H3.1K36M are incorporated into distinct chromatin regions.

**Fig. 6:**
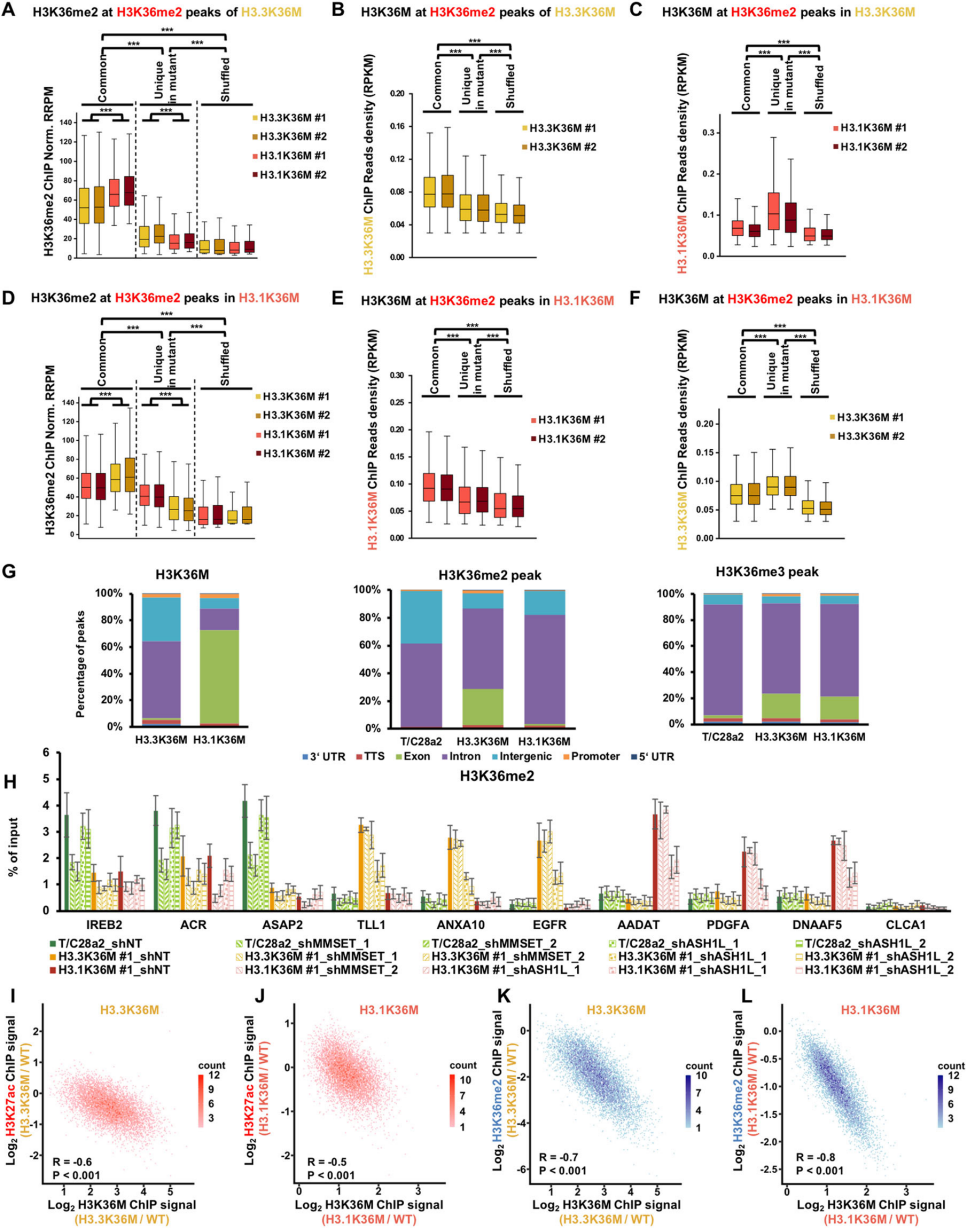
Epigenome is reprogrammed depending on the chromatic positioning of H3.3K36M and H3.1K36M. **A** The normalized read densities of H3K36me2 at common and unique H3K36me2 peaks in H3.3K36M-mutant cells. A set of shuffled peaks corresponding to the unique peaks was used to analyze whether the enrichment of the ChIP-seq signals was significant, when compared to background enrichment. ***p < 0.001. **B**, **C** The normalized read densities of H3.3K36M (**B**) and H3.1K36M (**C**) at common and unique H3K36me2 peaks in H3.3K36M-mutant cells. A set of shuffled peaks corresponding to the unique peaks was used to analyze whether the enrichment of the ChIP-seq signals was significant compared to background enrichment. ***p < 0.001. **D**–**F** Same as **A**–**C**, except the common and unique H3K36me2 peaks in H3.1K36M-mutant cells were analyzed. ***p < 0.001. **G** The percentages of H3K36M, H3K36me2, and H3K36me3 ChIP-seq peaks in genomic elements. The ChIP-seq results from independent H3.3K36M- and H3.1K36M-mutant clones were pooled. **H** ChIP-qPCR results of H3K36me2 after the depletion of MMSET and ASH1L in wild-type, H3.3K36M-, and H3.1K36M-mutant cells, respectively. The data are represented by the mean ± SD (*N* = 3 independent replicates). **I**, **J** Correlations between the changes in H3K27ac and H3K36M at active enhancers in wild-type cells that were within the H3K36me2 peaks in H3.3K36M- (**I**) and H3.1K36M- (**J**) mutant cells. Each dot indicates a single enhancer. *R*, correlation coefficient. The correlations were assessed by Pearson product moment correlation. **K**, **L** Correlations between the changes in H3K36me2 and H3K36M at active enhancers in wild-type cells that were within the H3K36me2 peaks in H3.3K36M- (**K**) and H3.1K36M- (**L**) mutant cells. Each dot indicates a single enhancer. *R*, correlation coefficient. The correlations were assessed by Pearson product moment correlation.

We speculated that the chromatin localizations of H3.3K36M and H3.1K36M led to the distinct reprogramming of H3K36 methylation. Therefore, we analyzed the correlations between the distribution of H3K36M-mutant protein and H3K36 methylation. We separated the H3K36 methylation peaks into two classes: peaks common in wild-type and mutant cells, and unique peaks in mutant cells. A set of shuffled peaks corresponding to the common peaks was used as a negative control. In H3.3K36M cells, both H3K36me2 and H3.3K36M were highly enriched at the common H3K36me2 peaks, followed by unique and shuffled peaks (Fig. 6A, B). The enrichment of H3K36me2 at the common peaks of H3.3K36M was lower in H3.3K36M cells than that in H3.1K36M cells. H3K36me2 at unique peaks of H3.3K36M was higher in H3.3K36M cells than H3.1K36M cells. Moreover, when compared with the enrichments in unique peaks, H3.3K36M was high and H3.1K36M was low at the common peaks (Fig. 6B, C). Vice versa, we observed the similar changes of H3K36me2 and H3K36M in H3.1K36M cells (Fig. 6D–F). Moreover, we detected the similar changes between H3K36me3 and H3K36M (Supplementary Fig. S7A–F). Notably, although the H3K36me2/me3 were high at the corresponding common peaks, they decreased in mutant cells (Supplementary Fig. S7G, H). We noticed that the H3K36me2 was redistributed to exons and H3.3K36M was enriched at introns in H3.3K36M cells (Fig. 6G). Interesting, H3.3K36M was enriched at introns and intergenic regions, while H3.1K36M was enriched mainly at exons, further supporting that H3.3K36M and H3.1K36M were distributed at different regions of chromatin. H3K36me2 was redistributed to introns and H3.1K36M was enriched at exons in H3.1K36M cells. This strong exclusive correlation was not detected at H3K36me3 peaks. The H3K36me2 methyltransferase MMSET, but not ASH1L, was inhibited by H3K36M mutation^13^, so we knocked-down MMSET and ASH1L to testify whether the redistribution of H3K36me2 would be blocked (Supplementary Fig. S7I, J). Three gene loci with redistributing H3K36me2 in either H3.3K36M (*TLL1*, *ANXA10*, and *EGFR*) or H3.1K36M cells (*AADAT*, *PDGFA*, and *DNAAF5*) were analyzed. Three gene loci (*IREB2*, *ACR*, and *ASAP2*) with decreased H3K36me2 in both mutant cells were analyzed as the “original” H3K36me2 sites. When MMSET was depleted, H3K36me2 at all of the tested “original” H3K36me2 sites was decreased in wild-type and mutant cells, and was unaffected at the redistributing sites (Fig. 6H). Depletion of ASH1L decreased H3K36me2 at all analyzed redistributing loci in the corresponding mutant cells, but showed less effects at the “original” H3K36me2 sites. These data suggest that depletion of ASH1L could, at least partially, inhibit the redistribution of H3K36me2 in H3K36M cells at the tested loci.

As in Fig. 4G, we used the active enhancers within H3K36me2 peaks in wild-type cells to directly analyze the effects of H3K36M. The correlations between the changes of H3K27ac and H3K36M were −0.6 and −0.5 in H3.3K36M and H3.1K36M cells, respectively (Fig. 6I, J). In addition, the correlations between the changes of H3K36me2 and H3K36M were −0.7 and −0.8 in H3.3K36M and H3.1K36M cells, respectively (Fig. 6K, L). In support of this observation, the correlations between H3K27ac and H3K36M were not detected when we used the enrichment of H3K36M with the other genotype (Supplementary Fig. S7K–M). Taken together, these results suggest that the genomic loci of incorporated H3K36M-mutant protein determine the reprogramming of epigenome.

### H3K36M-mutant protein decreases H3K36 methylation locally

**Fig. 7:**
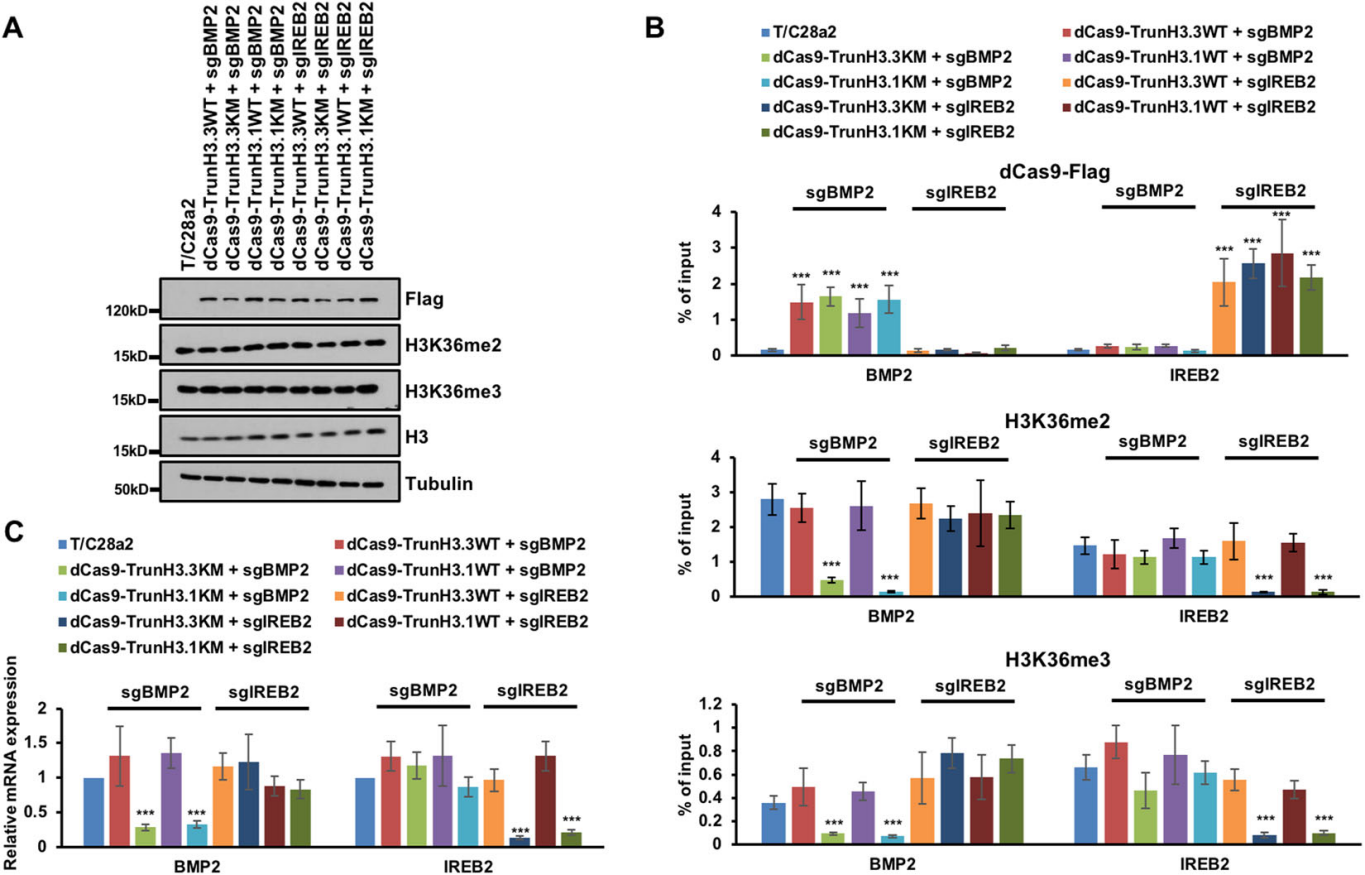
Targeting H3K36M-mutant protein to chromatin inhibited the H3K36 methylation locally. **A** Targeting truncated H3.3K36M and H3.1K36M to BMP2 and IREB2 loci didn’t change the total level of H3K36 methylation. T/C28a2 cells were infected with lentivirus to overexpress dCas9-fused H3.3WT, H3.3K36M, H3.1WT, and H3.1K36M with BMP2 and IREB2 sgRNAs respectively. Cell extracts were analyzed by western blotting using the indicated antibodies. **B** Targeting truncated H3.3K36M and H3.1K36M to BMP2 and IREB2 loci inhibited the H3K36 methylation locally. Upper: ChIP-qPCR analysis of dCas9-fused proteins at BMP2 and IREB2 loci. Middle: enrichments of H3K36me2 at BMP2 and IREB2 loci. Lower: enrichments of H3K36me3 at BMP2 and IREB2 loci. Data are mean ± SD (*N* = 3 independent replicates, ***p < 0.001). **C** The expressions of BMP2 and IREB2 decreased after targeting truncated H3.3K36M and H3.1K36M to BMP2 and IREB2 loci, respectively. The expressions of BMP2 and IREB2 were analyzed by quantitative RT-PCR and normalized to GAPDH. The expression levels of the genes in T/C28a2 cells were normalized as 1. Data are mean ± SD (*N* = 3 independent replicates, ***p < 0.001).

To evaluate the in cis effects, we utilized the CRISPR/dCas9 system to target H3K36M-mutant proteins to specific genomic loci. The full-length H3K36M-mutant protein could be incorporated into chromatin, so we expressed a dCas9 fused with 1–81 residues of H3, and then targeted to BMP2 and IREB2 with three and four sgRNAs, respectively. The targeting of H3.3K36M- and H3.1K36M-trancated protein (dCas9-Trun H3.3K36M and dCas9-Trun H3.1K36M) didn’t change the total levels of H3K36 methylation (Fig. 7A). The dCas9-fused proteins were enriched at the targeting locus, but not at the other loci we tested (Fig. 7B upper panel). More importantly, H3K36me2 and H3K36me3 were only decreased at the targeted loci (Fig. 7B middle and lower panel). Targeting of truncated wild-type H3 has no obvious effects on the H3K36 methylation locally. Moreover, the gene expressions of *BMP2* and *IREB2* were also inhibited when the truncated H3K36M-mutant proteins were targeted to their gene loci (Fig. 7C). H3.3K36M,R129E-mutant protein were expressed at a very low level and could still reduce the total levels of H3K36 methylation (Supplementary Fig. S8A)^20^. The levels of dCas9-Trun H3K36M were at least ten times more than the H3.3K36M,R129E (Supplementary Fig. S8B). It’s unlikely that the localized inhibition of truncated protein is due to its low expression level. Together, these data show that targeting either H3.3K36M or H3.1K36M decreases the H3K36 methylations locally.

## Discussion

We and others have previously found the H3.3K36M-mutant proteins reprogrammed the epigenome and gene expression through the inhibition of H3K36 methyltransferases^13–16^. In the present study, we uncover that the reprogramming of epigenome is not through random inhibition of methyltransferases, but through the incorporation sites of mutant histones. Moreover, the enhancers of mutant cells, which are not analyzed before, are reprogrammed depending on the loci of mutant histones. These founding may extend the oncohistones studies to the genetic difference beyond the proteins.

We notice that the H3K27me3 is not notably changed by western blot analysis (Fig. 1A). So, we try to dissect this by overexpression of H3K36M-mutant proteins. Indeed, with the higher amount of H3K36M expression in cells, there’s greater decreases of H3K36me2 and H3K36me3. Moreover, in the overexpression system, the H3K27me3 increased to a notable level by western blot. We don’t observe dramatical H3K27me3 changes by western blot analysis, but we do see the changes by ChIP-seq. At intergenic regions, the H3K27me3 ChIP-seq signal increased to ~1.4 times in mutant cells, when compared with wild-type cells (Supplementary Fig. S4A). H3K27me3 at gene body regions is not significantly changed in mutant cells (Supplementary Fig. S4B). We suspect that the total levels of H3K27me3 are not changed dramatically to be detected by Western blot. In line with this, Meers et al., replaced all histone H3 with H3K36R mutation in drosophila, they didn’t observe an increase of H3K27me3 by western blot^24^.

Studies by the Almouzni and Banaszynski’s groups suggest that H3.3S31 plays a role in the development^25^ and enhancer activation^26^. It’s also reported that S31 phosphorylation directly regulate the enzymatic activity of SETD2^27^. The truncated histones used in the recruitment experiments contain only 1–81 amino acids. The difference between these two mutant proteins is the S31 in H3.3 and A31 in H3.1, other amino acids are identical. We don’t detect the difference of inhibition on H3K36 methylation between truncated proteins when these proteins are targeted to the same loci by sgRNAs (Fig. 7B, C). The S31 is unlikely to contribute to the preferential determinants for chondroblastoma.

Cancer-associated histone mutations are often found in pediatric cancer patients, suggesting that the exact timing and developmental contexts are important for their carcinogenic effects^28^. Our data also support the idea that H3.3K36M and H3.1K36M change the expression levels of different sets of genes, and that the tumorigenic effects of H3.3K36M and H3.1K36M are largely dependent on the cellular context, in which the mutations occur. H3.3K27M mutations increased proliferation in human embryonic stem cells, but induced apoptosis in differentiated cells^29^. In addition, the time when the H3.3K27M mutation was introduced in mice had an especially pronounced effect on tumor occurrence^30,31^. In addition, H3.1K27M and H3.3K27M mutations reprogram the enhancer landscapes in a cell context-dependent manner^32^. This finding agrees with the idea that low levels of incorporation of H3K36M-mutant protein can reprogram the corresponding lysine methylation to new genomic loci.

The exact cell-of-original are not known yet, making it hard to exactly mimic the tumorigenesis of chondroblastoma. Although the cellular models we used here show the similar gene expression and epigenomic changes as the primary tumor tissues, it still may not precisely copy the tumorigenesis of chondroblastomas. More cell lines could be tested to verify this hypothesis. It will also be useful to address the detailed molecular mechanisms, if we could develop the mouse models or found the cell-of-original of chondroblastomas. To further understand the impact of H3.3K36M-mutant protein on the corresponding H3K36 methyltransferases in vivo, there is an urgent need for the development of antibodies suitable for H3K36 methyltransferase ChIP-seq experiments to compare the genomic distributions of these enzymes, with those of H3K36M-mutant proteins. In this way, the effects of H3K36M-mutant proteins on the reprogramming of histone methyltransferases could be elucidated by the findings of which methyltransferases are, if so, redistributed.

## Materials and methods

### Cell lines

HEK293T cell was purchased from ATCC and cultured in DMEM supplemented with 10% FBS for lentiviral production. T/C28a2 cell, which was the immortalized human juvenile costal chondrocyte, was cultured in DMEM supplemented with 10% FBS. All cell lines were tested negative for mycoplasma using a qPCR-based testing with mixed primers.

### Knock-in of K36M at HIST1H3D using CRISPR/Cas9 system

The sgRNA: CGTGCCGGGCCGGTAACGGT was cloned into pSpCas9(BB)-2A-Puro V2.0 plasmid. To generate the K36M mutation, a single-strand DNA, listed below, was co-transfected with the sgRNA plasmid into T/C28a2 cells using Nucleofector Kit V (Lonza), according to previously published protocol^33^. Cells were grown under puromycin selection (2 μg/ml) for 2 days before switched into normal growth medium. Around 500 single clones were analyzed to identify the correct knock-in. Genomic DNA was amplified and sequenced with the primer pair: forward, TAGTCACTCGCTTGGCGTG; reverse, GTGGGCGTCTCCACCAATC. Two independent clones harboring the heterozygotic K36M mutation at *HIST1H3D* gene were identified.

Single-strand donor DNA used:

GTTTGCGAATCAGCAGCTCGGTCGACTTCTGGTAGCGGCGGATCTCGCGCAGAGCCACCGTGCCGGGCCGGTAACGATGAGGCTTCATCACGCCGCCGGTGGCTGGAGCGCTCTTTCGAGCAGCCTTGGTGGCCAGCTGCTTGCGTGGCG.

### In vitro differentiation of T/C28a2 cells

The chondrogenic differentiation was performed as previous described with modifications. In brief, 10 μl of cells was seeded as a micromass at a density of 2 × 10^7^/ml. Micromass was then cultured in differentiation medium, DMEM supplemented with 5% FBS, 50 μg/ml ascorbic acid, 10 mM β-glycerol-phosphate, and 1× insulin–transferrin–selenium, for 7 days. Cells were feed with differentiation medium every 3 days^34^. The micromasses were then fixed with 10% neutral-buffered formalin for 10 min before staining with Alcian blue (3% acetic acid and 1% Alcian blue) for 2 h. After several washes with double-distilled water, cells were photographed under a microscope (4× objective, Olympus).

### Cell proliferation and colony formation assays

For cell proliferation, cells were plated into each well of 96-well plate at a density of 3 × 10^3^ in 100 μl of growth medium. Relative number of cells was determined by the cell titer blue assay kit (Promega) according to the manufacturer’s instructions.

For colony formation, 400 cells were seeded to each well of a six-well plate. The cells were then cultured for 3 weeks before crystal violet staining.

### Detection of apoptosis

Different cells were treated with 0.5 μM staurosporine for 3 h before being stained by Annexin V-FITC/PI staining kit (Sangon Biotech), according to the manufacturer’s instructions. The stained cells were then analyzed by a flow cytometry at the core facility of Life Sciences Institute, Zhejiang University.

### MNase digestion

A total of 4 × 10^6^ cells were collected and washed in nuclear extraction buffer A (85 mM KCl, 10 mM Tris pH 7.5, 0.2 mM spermidine, 0.2 mM EDTA, 160 mM sucrose, and 250 μM PMSF) for 5 min. The samples were then lysed in nuclear extraction buffer B (buffer A + 0.1% NP40) for 5 min to extract nuclei. Nuclei were washed in digestion buffer (50 mM Tris pH 7.5, 20 mM KCl, 0.32 M sucrose, 4 mM MgCl_2_, and 3 mM CaCl_2_), and digested with micrococcal nuclease (2000 units/ml) for different time points: 0, 1, 2.5, 5, 10, and 20 min, at 37°. Equal volume of stop buffer (0.2% SDS and 10 mM EDTA) was added at 37° for 10 min. The samples were incubated with 10 μg/ml RNase A at 37° for 30 min, and then 100 μg/ml proteinase K at 55° for 1 h. DNA was purified with phenol/chloroform/isoamyl alcohol.

### Microsatellite instability (MSI) assay

MSI assays were conducted as previously described with modifications^35^. Briefly, genomic DNA was isolated by Gentra Puregene Cell Kit (Qiagen) and subjected to PCR analysis with denaturing acrylamide gel.

### Oligonucleotide

See Supplementary Table S1 for detailed primers for RT-PCR and ChIP-qPCR.

### Antibodies

Antibodies against H3K27me3 (Cat. #9733), H3K36me2 (Cat. #2901), H2A (Cat. #12349), and H2AK119Ub (Cat. #8240) were purchased from Cell Signaling. Antibodies against H3K9me3 (Cat. #ab39161) were purchased from Abcam. H3K36me3 antibodies were purchased from Active Motif (Cat. #61101). Antibodies against H3K36M (Cat. #31-1085-00) were purchased from RevMAb Biosciences. Antibodies against H3K4me3 (Cat. #07-473) were purchased from Millipore. Antibodies against histone H3 were previously described^36^.

### Reverse transcription-PCR

Total RNA was extracted using RNeasy plus kit (Qiagen) according to the manufacturer’s instructions. A total of 0.5 μg of total RNA, random hexamers, and Superscript III Reverse Transcriptase were then used to synthesize cDNAs. Real-time PCR was carried out using SYBR Green PCR Master Mix (Bio-Rad). The expression levels of GAPDH were determined as a control to normalize the expression of target genes.

### ChIP-seq

ChIP-seq was performed as previously described^37^. Cells were crosslinked by 1% formaldehyde for 10 min, and then quenched in 125 mM glycine for 5 min. After washing with cold TBS twice, cells were incubated in lysis buffer (10 mM Tris-HCl, pH 7.5, 10 mM NaCl, and 0.5% NP40) for 10 min on ice. Nuclei were washed and resuspended in MNase digestion buffer (20 mM Tris-HCl, pH 7.5, 15 mM NaCl, 60 mM KCl, and 1 mM CaCl_2_) supplemented with 1000 units of MNase (NEB) and incubated at 37 °C with continuous mixing for 20 min. The reaction was stopped by adding of 2XSTOP buffer (100 mM Tris-HCl, pH8.1, 20 mM EDTA, 200 mM NaCl, 2% Triton X-100, and 0.2% sodium deoxycholate), followed by sonication for 10 min (30 s on/30 s off). For spike-in ChIP-seq, the MNase digested chromatin from drosophila S2 cells was added to 1–5% of total chromatin. The chromatin was incubated with antibodies at 4 °C overnight and then subjected to 30 μl of protein G-magnetic beads for additional 2 h. The beads were extensively washed and bound DNA was eluted with elution buffer (10 mM Tris-HCl, pH8.0, 10 mM EDTA, 150 mM NaCl, 5 mM DTT, and 1% SDS) and reverse-crosslinked at 65 °C overnight. DNAs were purified using Min-Elute PCR purification kit (Qiagen) after treatment of proteinase K and RNase A. The H3K36M ChIP-seq was performed without spike-in. Two biological repeats were performed for each ChIP-seq. The correlations between two biological repeats were summarized in Supplementary Table S2.

Sequencing libraries were prepared using TruPrep DNA library prep kit (Vazyme) following the manufacturer’s instructions. The library was sequenced on an Illumina HiSeq Xten platform with pair-end read of 150 bp.

### ChIP-seq data analysis

Sequencing reads were cleaned with trim-galore and aligned to the human genome, hg19, using bowtie2^38^. PCR duplicated reads were removed by SAMtools^39^. ChIP-seq peaks were identified by MACS2^40^ with the parameter of narrow peak for H3K36M peaks, and broad peak calling for H3K36 and H3K27 methylations. BEDTools^41^ and in-house shell program were used to calculate the genome coverage and reads density. The promoters were defined as 2 kb upstream and 500 bp downstream of TSS, which is determined by NCBI I RefSeq. Enhancers were defined as genomic loci that were overlapped with H3K4me1 peaks and were not overlapped with H3K4me3 peaks or annotated promoters.

For spike-in ChIP-seq results, the sequencing reads were aligned and normalized according to previous published procedures^42^. In brief, human (hg19) and drosophila (dm6) genome sequences were combined to produce a reference genome. Cleaned sequencing reads were aligned to the reference genome by bowtie2 with default parameters. The mapped reads were then separated into human and drosophila reads, where the reads mapped to the drosophila genome were used to determine the normalization factor. The number of reads in each sequencing results was counted and normalized by the normalization factor.

### RNA-seq and data analysis

RNA-seq libraries were prepared and sequenced with a BGISEQ-500 instrument. In brief, the poly-A-containing mRNA was purified by poly-T oligo-attached magnetic beads, and then fragmented by divalent cations under elevated temperature. The cleaved RNA was reverse transcript into cDNA with random primers, followed by second strand cDNA synthesis with DNA polymerase I and RNase H. These single “A” base added cDNAs were ligated to adapter, amplified, and then purified as single-strand DNA circle, which was used to generated DNA nanoballs (DNBs) by rolling circle replication PCR. Then DNBs were sequenced with pair-end read of 100 bp on the BGISEQ-500 platform.

Sequence reads were cleaned with trim-galore and then aligned to the human genome hg19 using STAR^43^. The mapped reads were annotated to Refseq genes using TopHat^44^, and the FPKM of genes was calculated by Cufflinks^45^. Cuffdiff was used to identify and analyze the differential gene expression using false discovery rate (FDR) < 0.05 as the cutoff.

### Statistics

The large sample sizes were corrected for multiple testing by Dunnett’s test.

### CRISPR/dCas9 experiment

In brief, the cloning of the dCas9-fused truncated histones was based on the pHR-SFFV-dCas9-BFP-KRAB plasmid, which was a gift from Stanley Qi and Jonathan Weissman (Addgene plasmid # 46911). The 1–81 amino acid residues of wild-type H3.1, wild-type H3.3, H3.1K36M, and H3.3K36M-mutant histones were cloned into the C-terminal of dCas9 in the expression vector, respectively. In addition, a Flag tag was added to the C-terminal end replacing the BFP-KRAB sequence. SgRNAs against BMP2 and IREB2 (Supplementary Table. S1) were cloned into the pLentiGuide plasmid, which was a gift from Paul Khavari (Addgene plasmid # 117986). Lentivirus expressing the dCas9 proteins and sgRNAs were produced separately in the 293T cells, and were then used to infect T/C28a2 cells. After infection, T/C28a2 cells were selected with 2 μg/ml puromycin for additional 2 days. Cells were then collected for western blot, RT-PCR, and ChIP-qPCR analysis, as described before.

### Accession numbers

Previous data used for H3K36M ChIP-seq in H3.3K36M cells: GSE75234.

Raw data have been deposited in the GEO database with the series accession GSE130858.

## Supplementary information

Figure S1

Figure S2

Figure S3

Figure S4

Figure S5

Figure S6

Figure S7

Figure S8

Supplementary figures

Supplementary figure legend

Supplementary table
