# Supplementary figures and images for: The incorporation loci of H3.3K36M determine its preferential prevalence in chondroblastomas
Source: Cell Death Dis. 2021 Mar 24;12(4):311. doi: 10.1038/s41419-021-03597-9 (PMC7991640; doi:10.1038/s41419-021-03597-9)

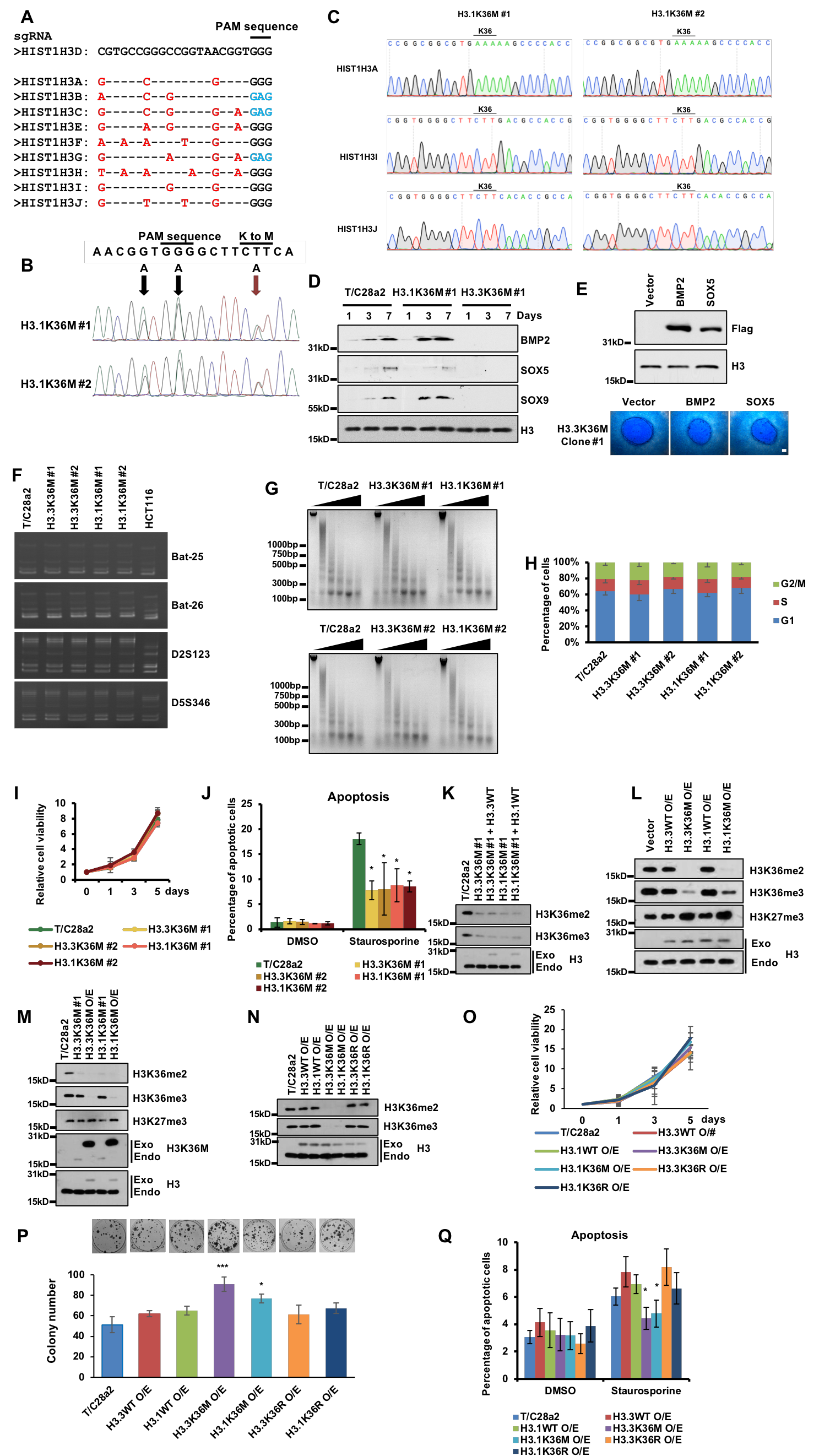

Supplement: Supplementary file 1 — Figure S1 [file 41419_2021_3597_MOESM1_ESM.tiff]

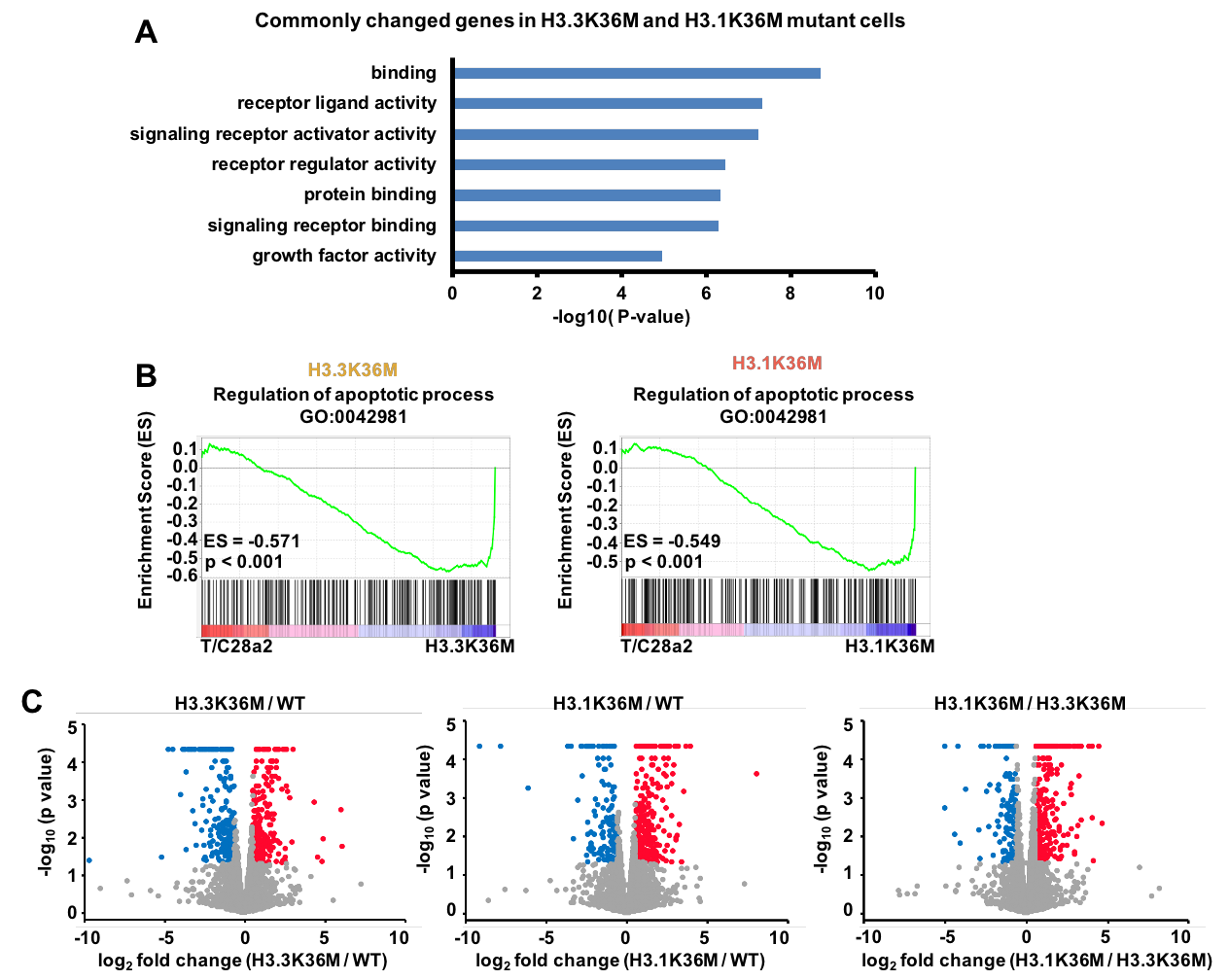

Supplement: Supplementary file 2 — Figure S2 [file 41419_2021_3597_MOESM2_ESM.tif]

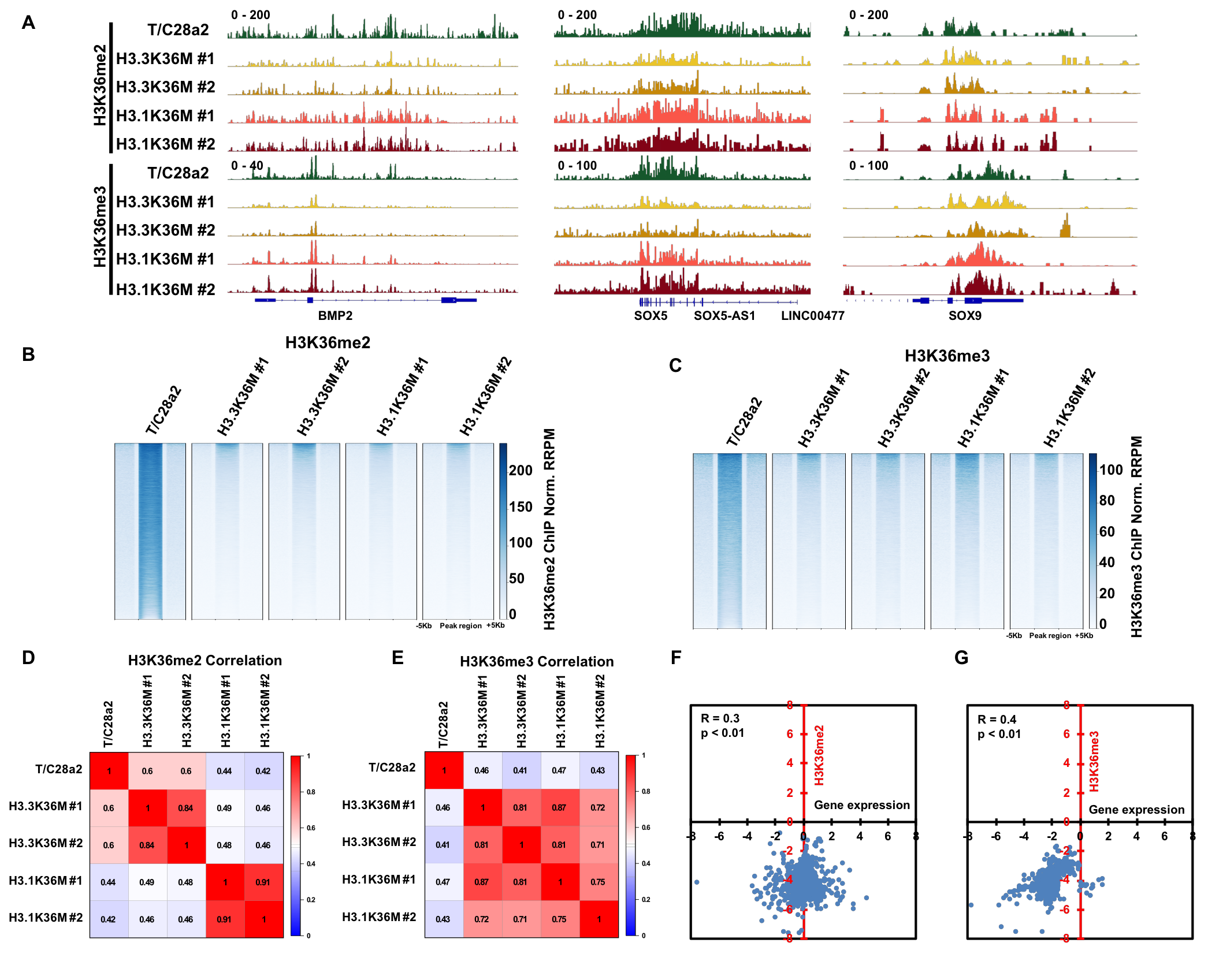

Supplement: Supplementary file 3 — Figure S3 [file 41419_2021_3597_MOESM3_ESM.tif]

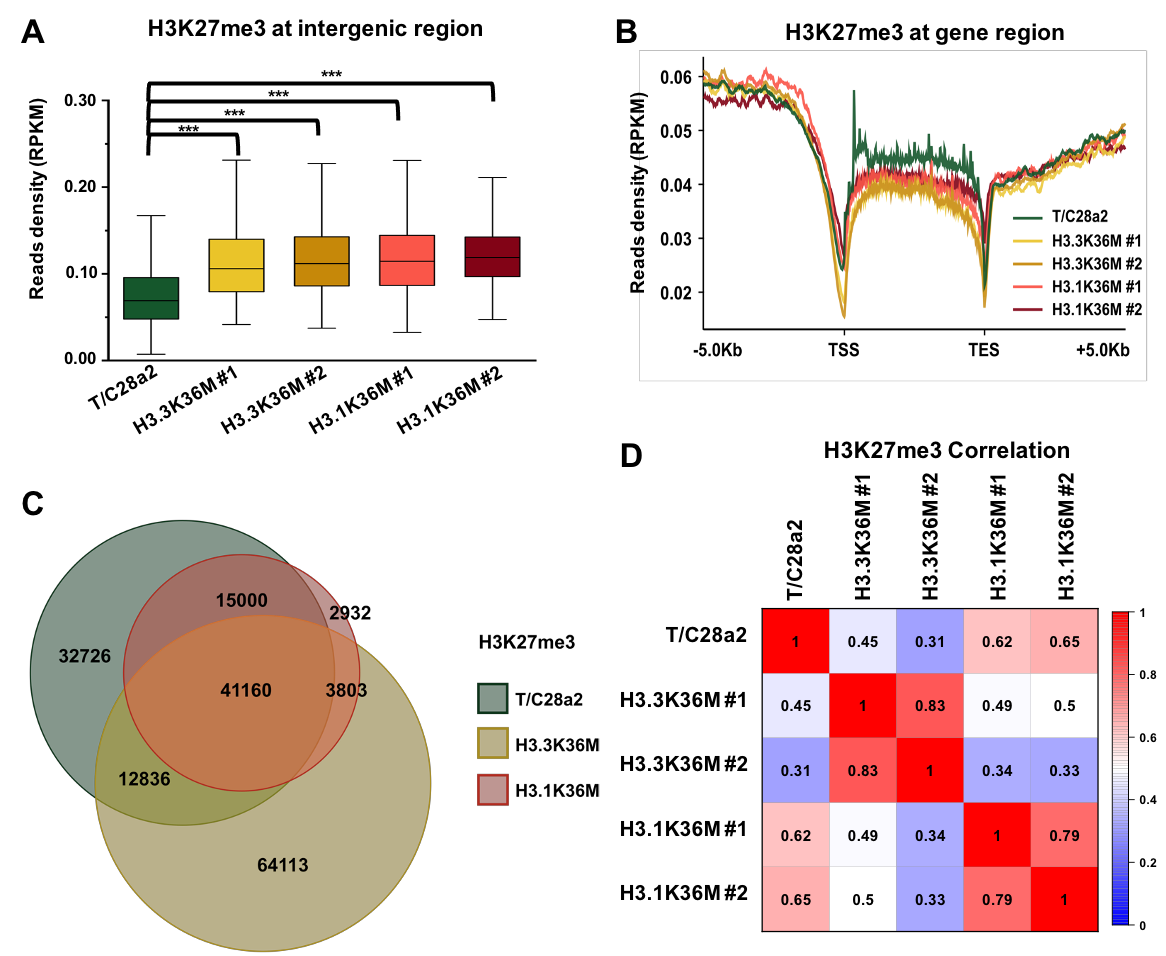

Supplement: Supplementary file 4 — Figure S4 [file 41419_2021_3597_MOESM4_ESM.tif]

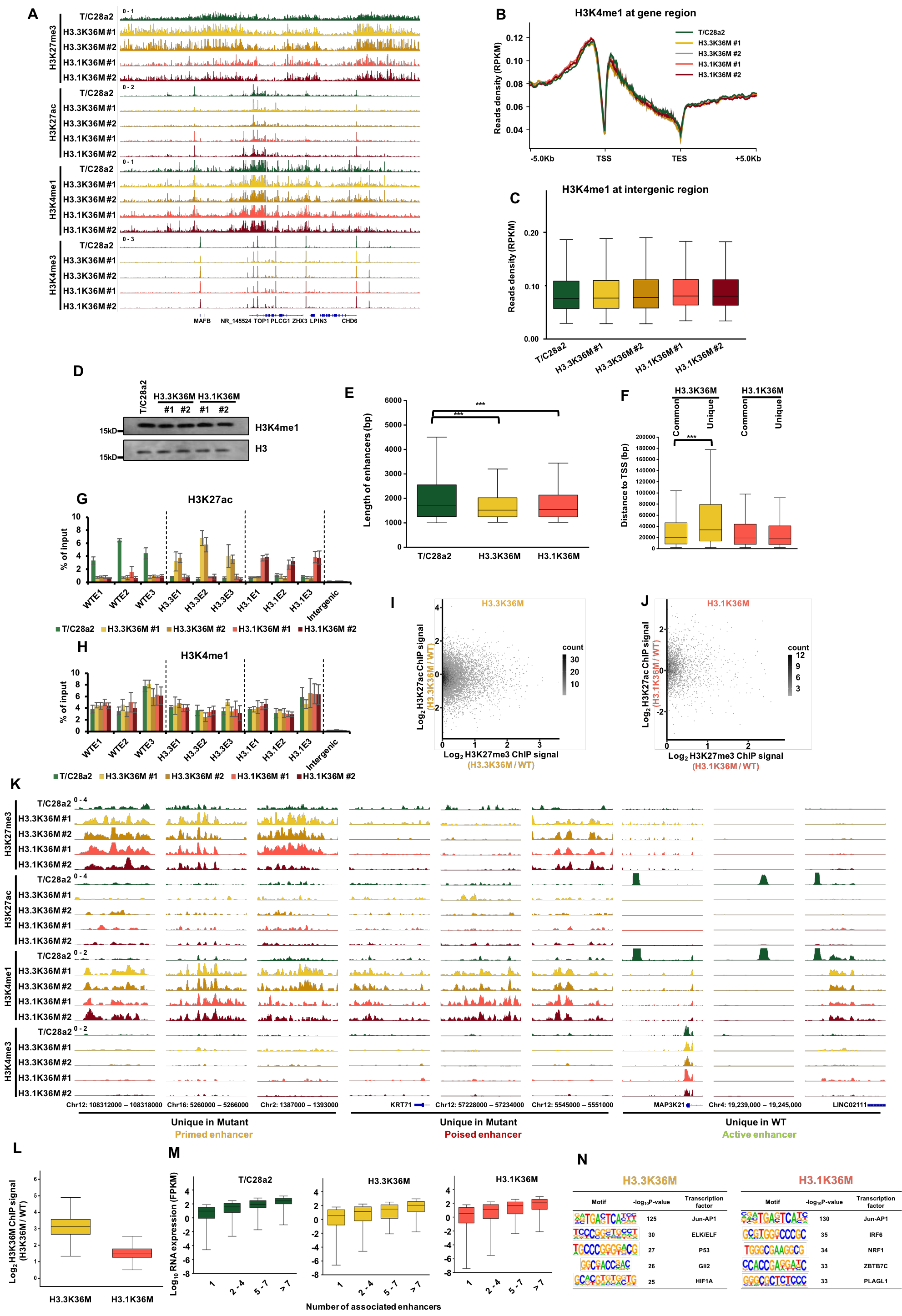

Supplement: Supplementary file 5 — Figure S5 [file 41419_2021_3597_MOESM5_ESM.tif]

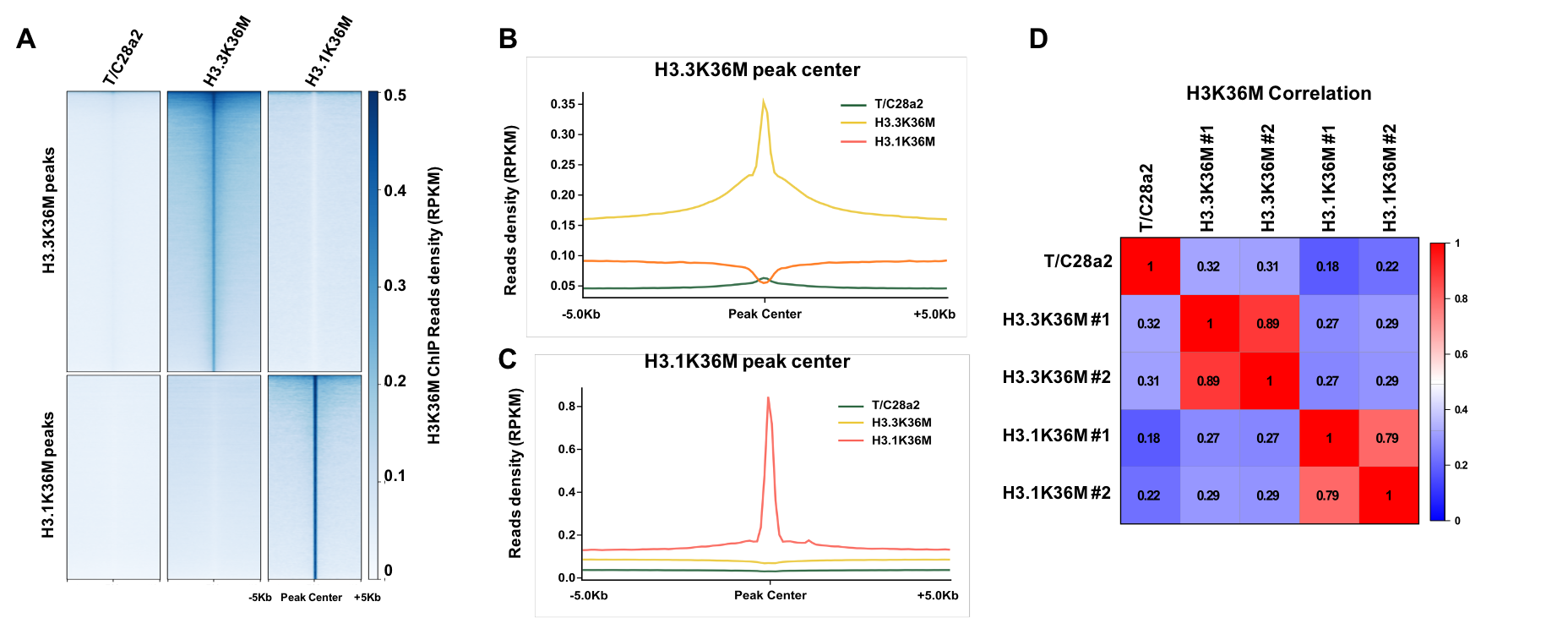

Supplement: Supplementary file 6 — Figure S6 [file 41419_2021_3597_MOESM6_ESM.tif]

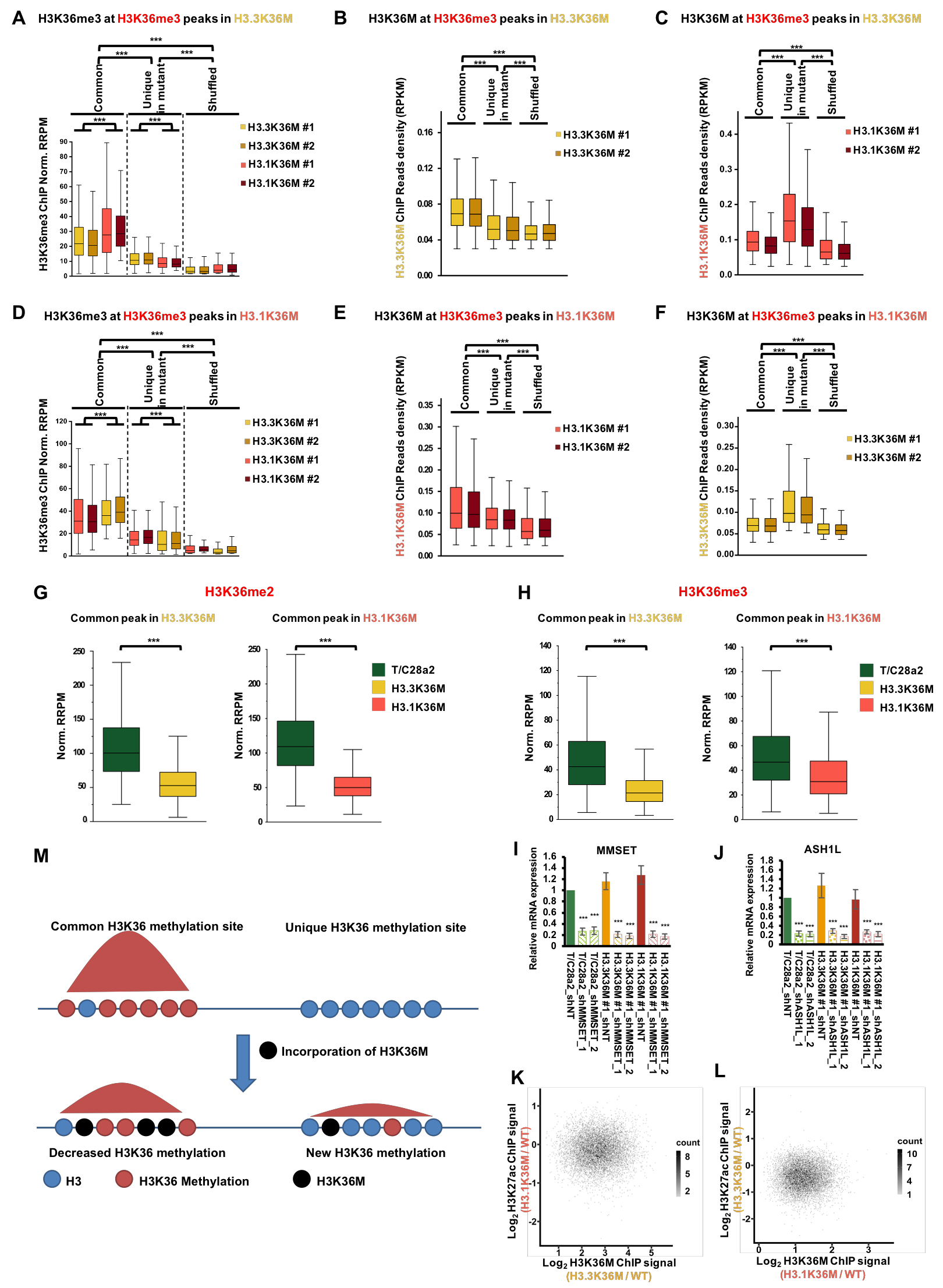

Supplement: Supplementary file 7 — Figure S7 [file 41419_2021_3597_MOESM7_ESM.tif]

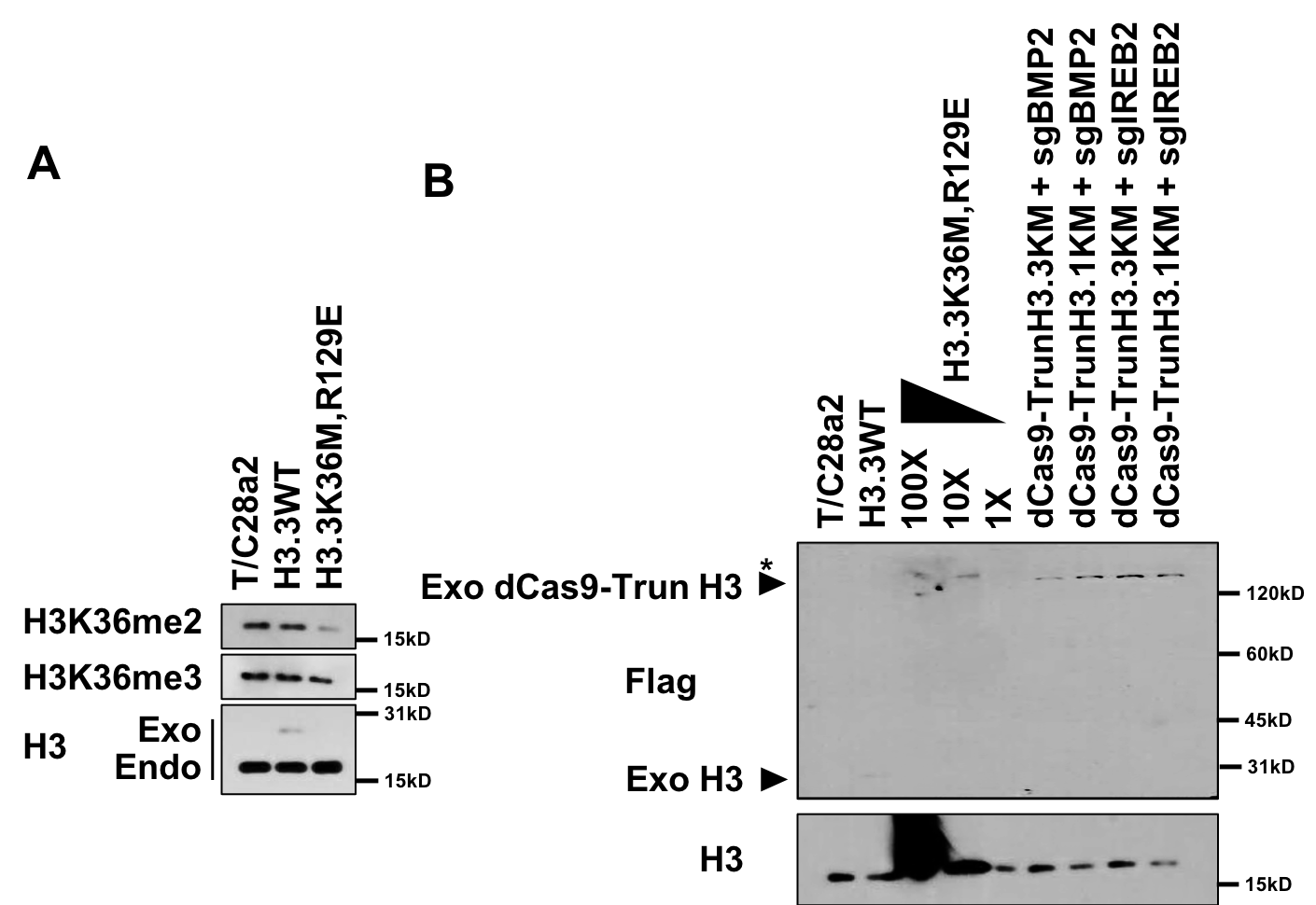

Supplement: Supplementary file 8 — Figure S8 [file 41419_2021_3597_MOESM8_ESM.tif]

**Figure S1**

**
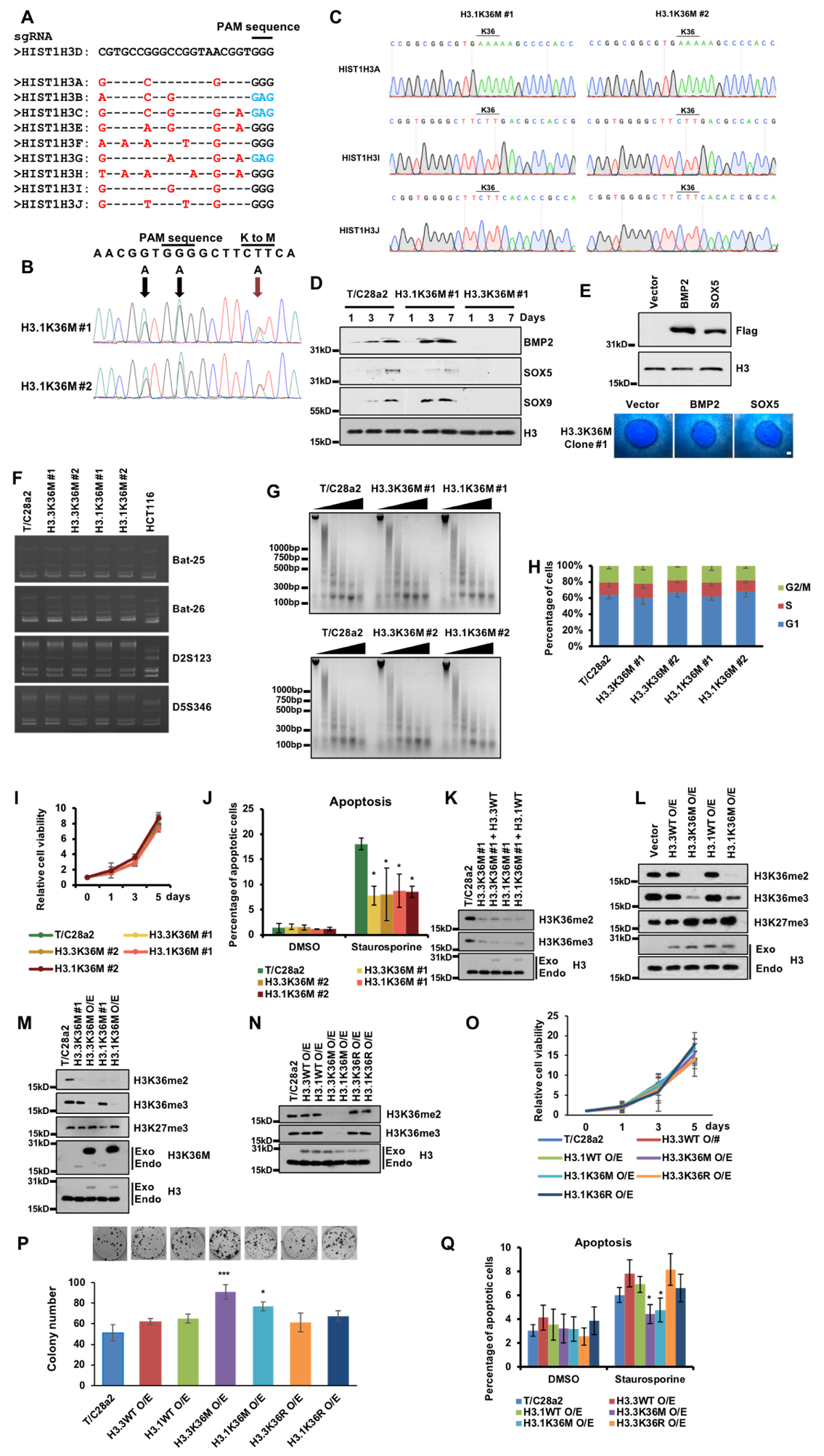
**

**Figure S2**


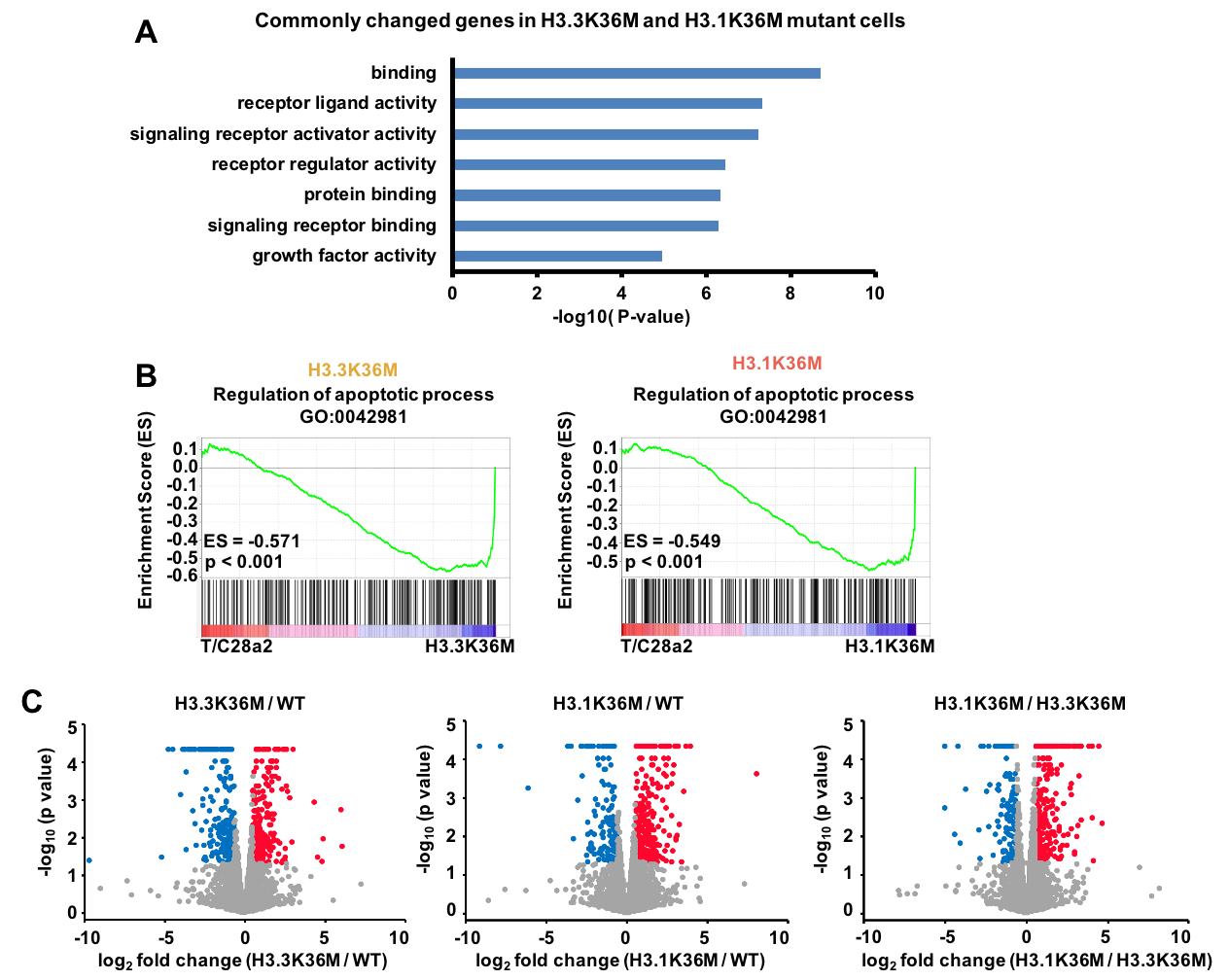


**Figure S3**
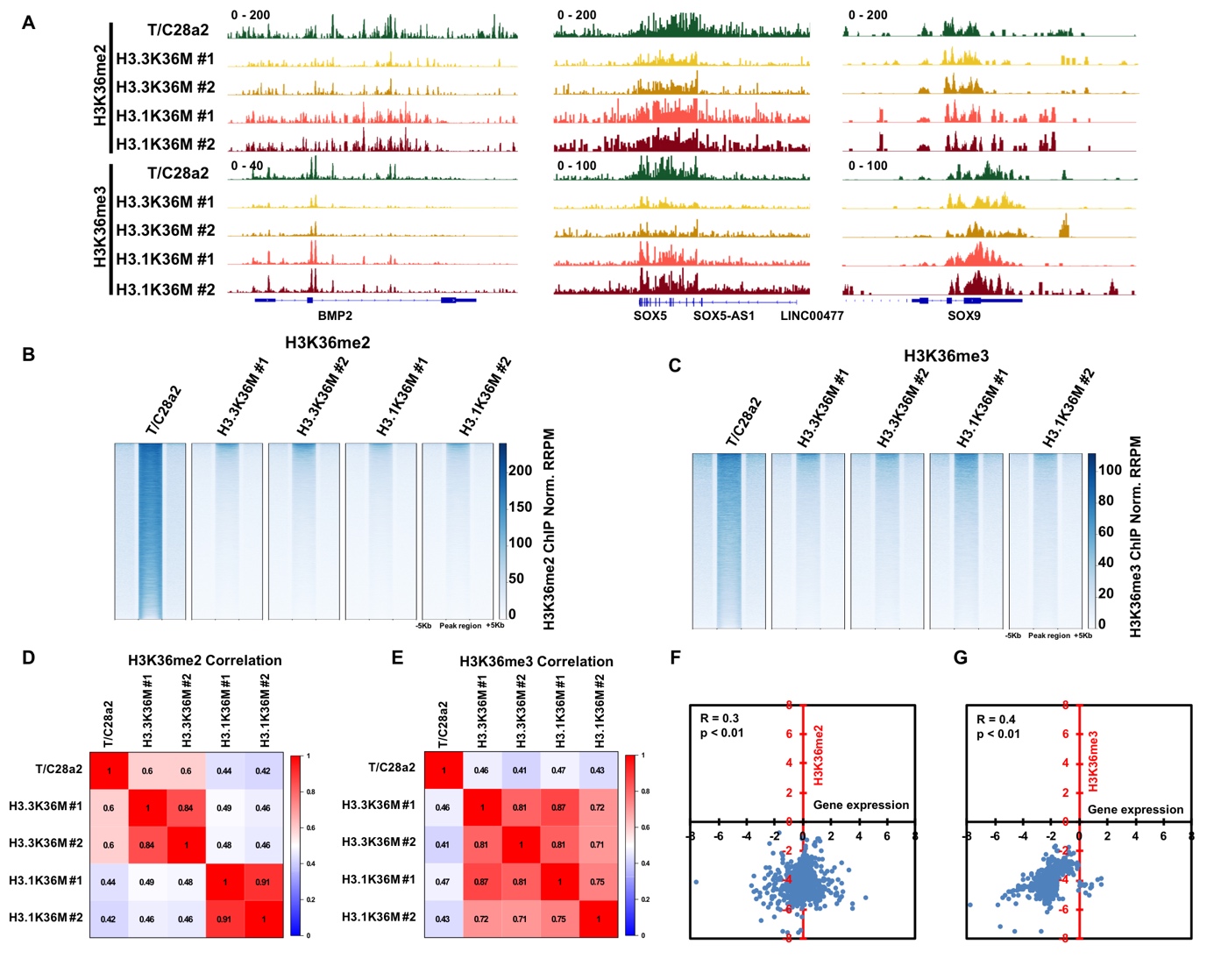


**Figure S4**
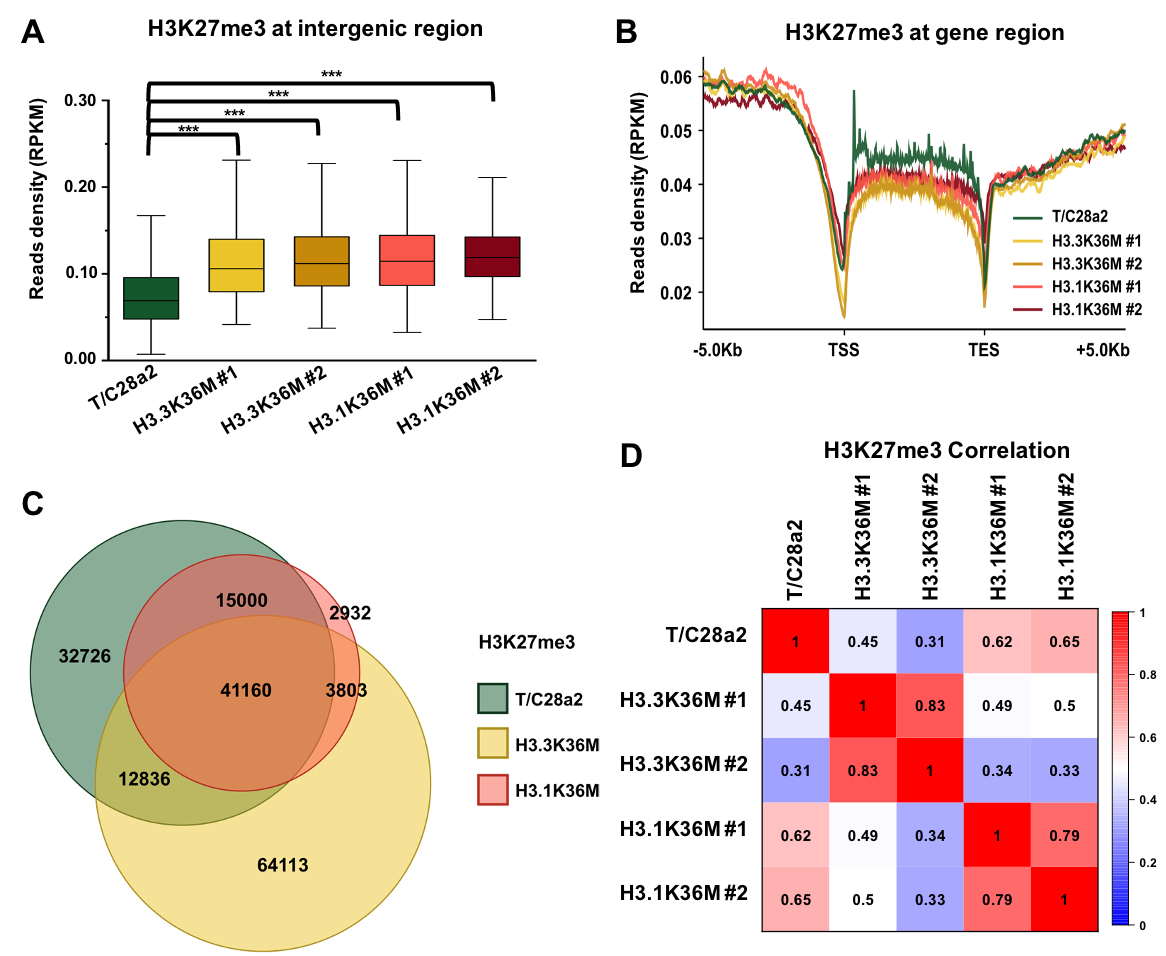


**Figure S5**
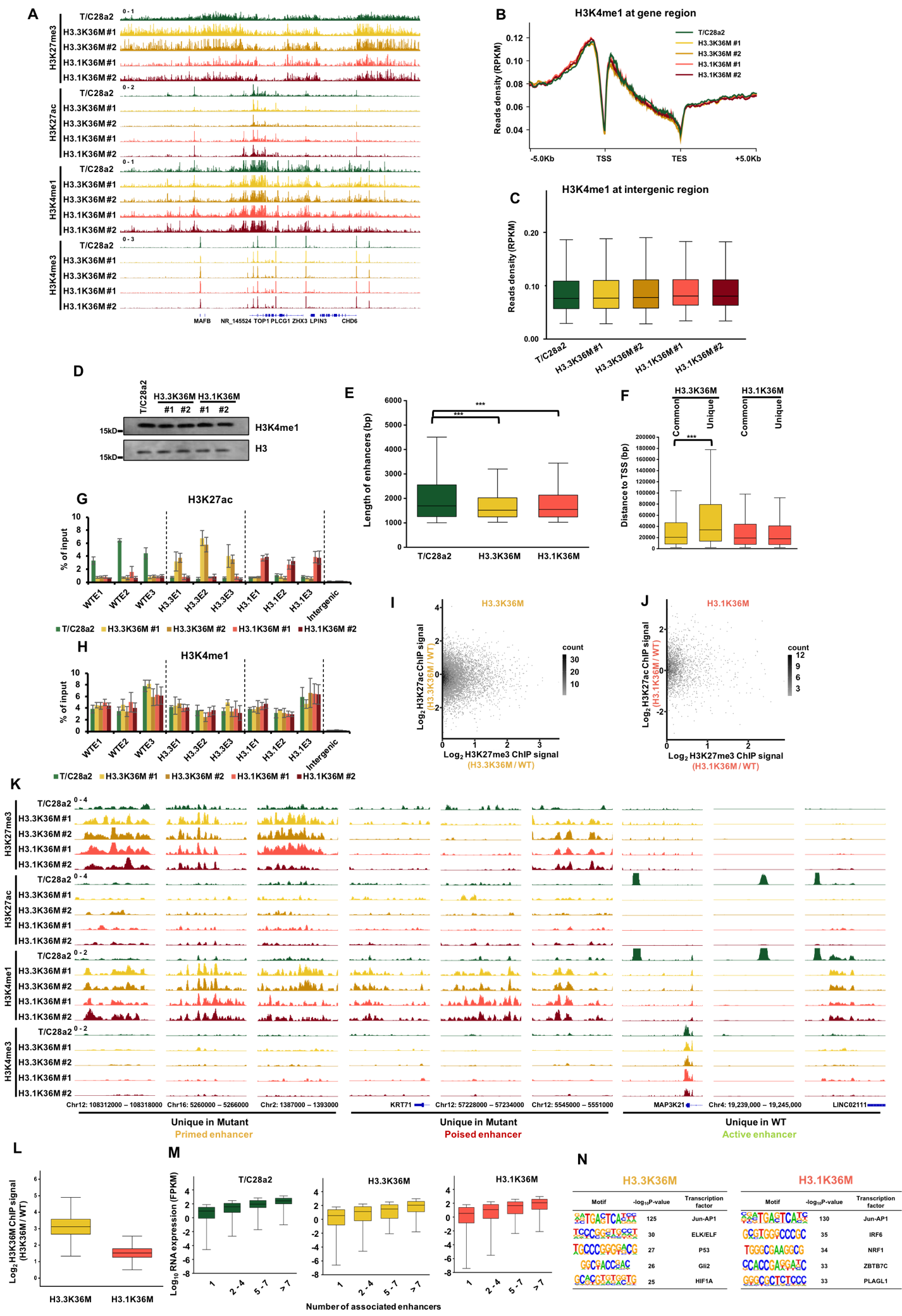


**Figure S6**
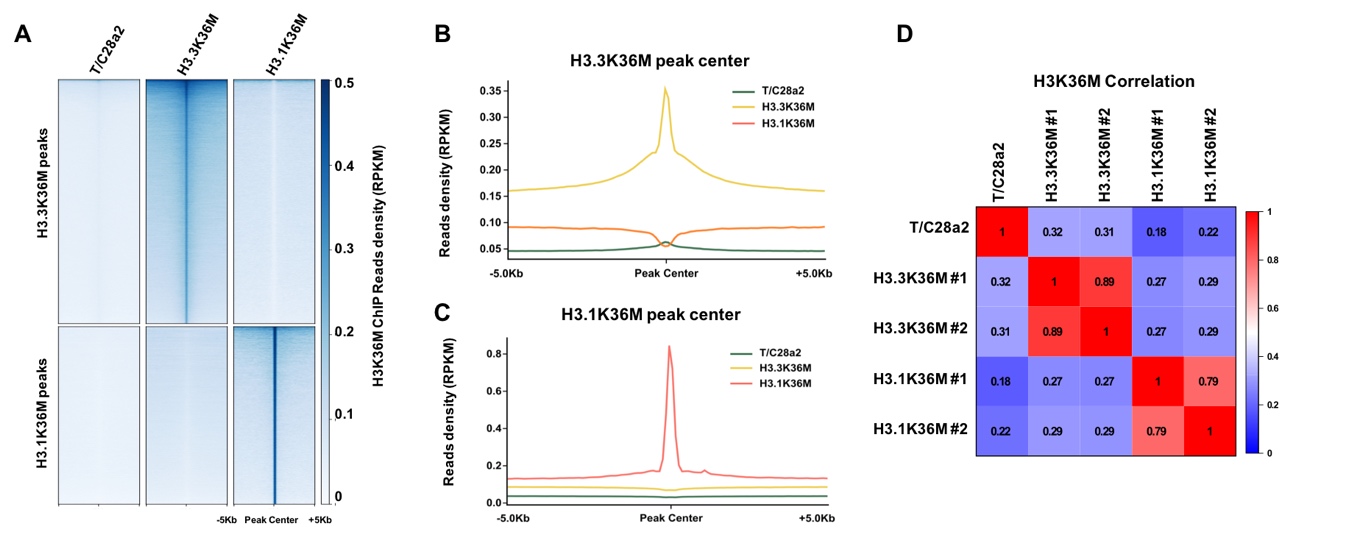


**Figure S7**
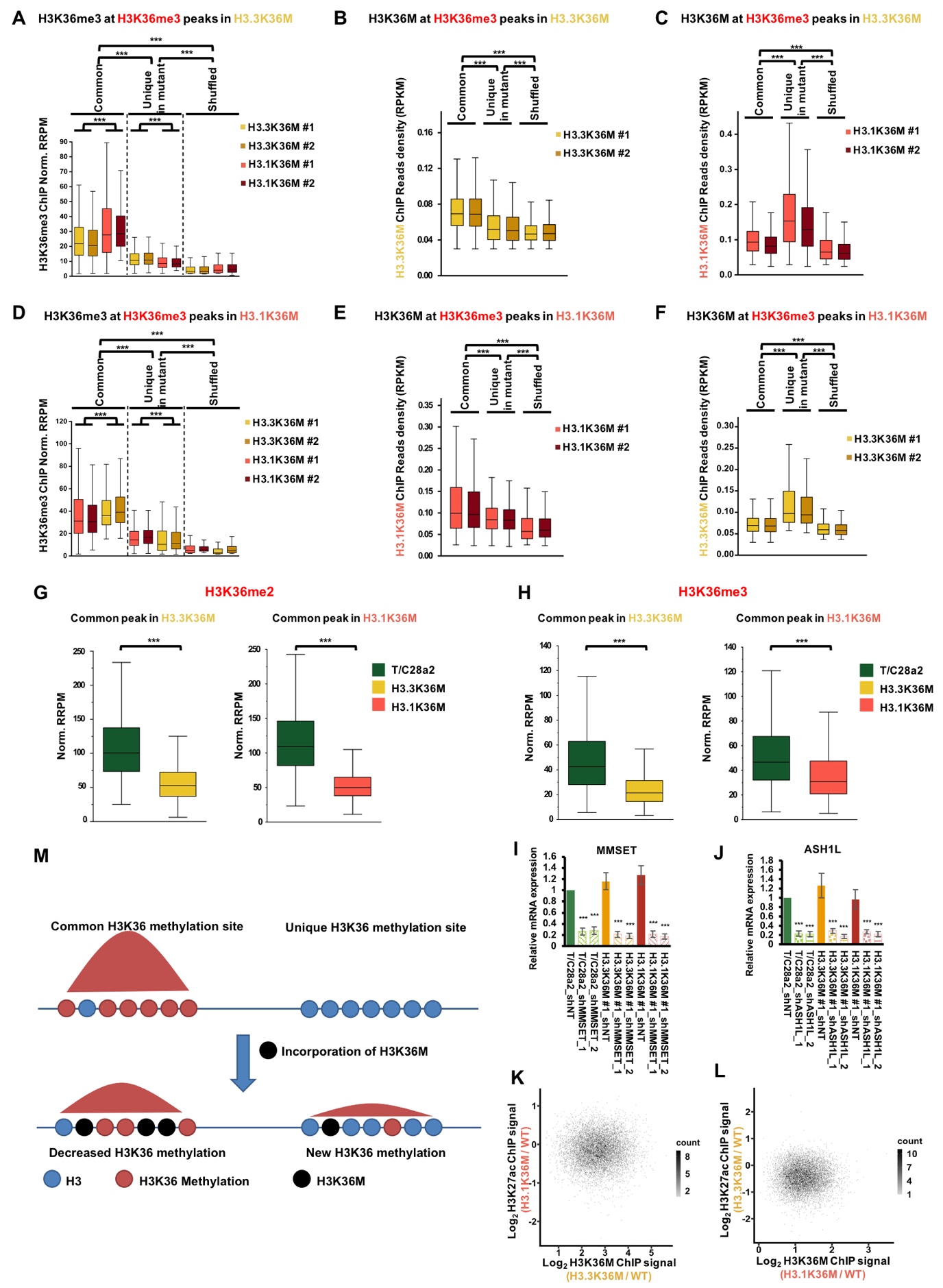


**Figure S8**
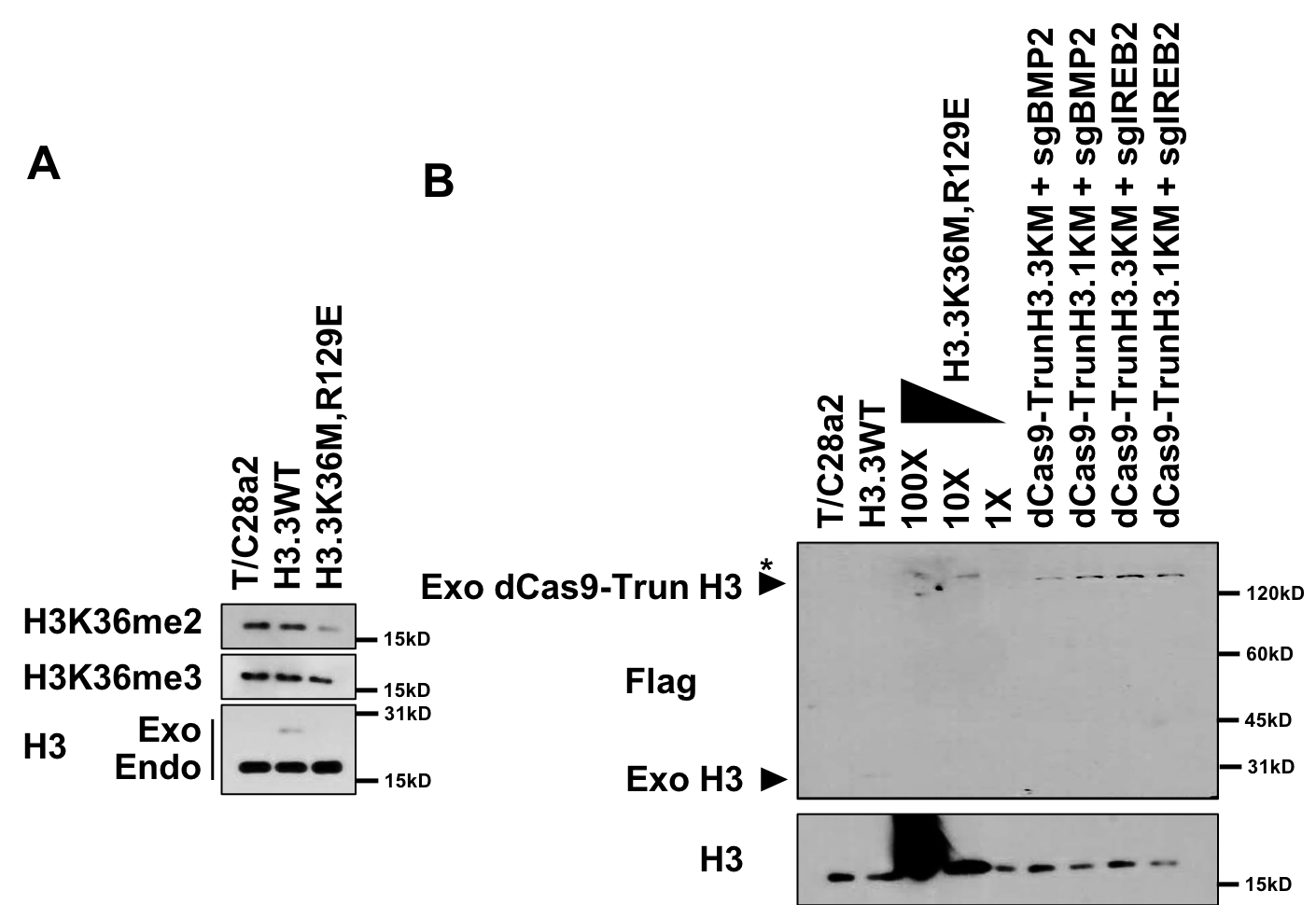

Supplement: Supplementary file 9 — Supplementary figures [file 41419_2021_3597_MOESM9_ESM.docx]
