# Supplementary material for: The incorporation loci of H3.3K36M determine its preferential prevalence in chondroblastomas
Source: Cell Death Dis. 2021 Mar 24;12(4):311. doi: 10.1038/s41419-021-03597-9 (PMC7991640; doi:10.1038/s41419-021-03597-9)
Supplement: Supplementary file 10 — Supplementary figure legend [file 41419_2021_3597_MOESM10_ESM.docx]

**Supplementary figure legends**

**Figure S1. H3.1K36M mutant cells show normal chondrogenic differentiation.**

(A) The DNA sequence at the sgRNA targeting site. The PAM sequence is indicated. Red nucleotides indicated the different nucleotides compared with *HIST1H3D* gene and blue nucleotides indicated the sites without PAM sequence. The sequence is from 3’ end to 5’ end of indicated genes.

(B) One allele of *HIST1H3D* gene was mutated to K36M in two independent T/C28a2 cell clones. The sanger sequencing results of *HIST1H3D* gene locus, which was amplified from genomic DNA, were shown. The red arrow indicated the K36M mutation site and black arrows showed the synonymous mutations which protected the donor DNA from cutting by the CRISPR/Cas9. The sequencing result is from 3’ end to 5’ end of *HIST1H3D* gene.

(C) The sanger sequencing results of three potential off-target sites in two H3.1K36M knock-in clones.

(D) Western blot showing the protein levels of BMP2, SOX5 and SOX9 in wild-type and mutant cells during differentiation.

(E) Over-expression of BMP2 or SOX5 alone didn’t rescue the differentiation defect in H3.3K36M mutant cells. Upper panel, Western blot showing the protein levels of over-expressed BMP2 and SOX5. Lower panel, alcian blue staining of differentiated micromasses. Scale bars: 50 μm.

(F) H3.3K36M and H3.1K36M mutations showed no obvious effect on the microsatellite instability. Genomic DNA was purified and subjected to PCR analysis for four microsatellite markers. HCT116 cells which were known with microsatellite instability were used as positive control.

(G) Micrococcal nuclease (MNase) digestion patterns of the chromatin in wild type, H3.3K36M and H3.1K36M cells. The digestion time used in each cell lines was 0, 1, 2.5, 5, 10, and 20 mins, respectively.

(H) H3.3K36M and H3.1K36M mutations did not affect the cell cycle. Cell cycle were analyzed by PI staining with FACS sorting. Data represented the mean ± SD (N = 3 independent replications).

(I) H3.3K36M and H3.1K36M mutations did not apparently affect the cell proliferation. Data represented the mean ± SD (N = 3 independent replications).

(J) Annexin V-positive cells were quantified with fluorescence-activated cell sorting after DMSO or staurosporine treatment. Data represented the mean ± SD (N = 3 independent replications, * p < 0.05)

(K) Over-expression of H3.3 and H3.1 wild-type proteins in H3.3K36M and H3.1K36M knock-in cells didn’t rescue the reduction of H3K36 methylation, respectively.

(L) H3.3K36M and H3.1K36M over-expression increased the H3K27me3 levels in chondrocyte cell lines. T/C28a2 cells were infected with lenti-virus to over-express H3.3WT, H3.3K36M, H3.1WT, and H3.1K36M. Cell extracts were analyzed by Western blotting using the indicated antibodies. O/E, over-expression.

(M) Western blot showing the histone marks in knock-in cells and over-expression cells. Cell extracts were analyzed by Western blotting using the indicated antibodies. O/E, over-expression.

(N) Over-expression of H3.3K36M and H3.1K36M but not H3.3K36R and H3.1K36R decreased the global H3K36 methylation. Cell extracts were analyzed by Western blotting using the indicated antibodies. O/E, over-expression.

(O) H3.3K36M, H3.1K36M, H3.3K36R and H3.1K36R over-expression did not apparently affect the cell proliferation. Data represented the mean ± SD (N = 3 independent replications). O/E, over-expression.

(P) Over-expression of H3.3K36M and H3.1K36M mutant proteins increased colony formation ability respectively. Results represented the mean ± SD (N = 3 independent replications, ***p < 0.001, *p < 0.05). O/E, over-expression.

(Q) Annexin V-positive cells were quantified with fluorescence-activated cell sorting after DMSO or staurosporine treatment. Data represented the mean ± SD (N = 3 independent replications, * p < 0.05). O/E, over-expression.

**Figure S2. Genes are expressed differently in wild-type, H3.3K36M and H3.1K36M cells.**

(A) GO analysis results of both changed genes in H3.3K36M and H3.1K36M mutant cells.

(B) GSEA analysis of the enrichment of both changed genes in the regulation of apoptotic process, GO: 0042981. The enrichment plot is shown at the top. GSEA was carried out by computing genes changed in both H3.3K36M and H3.1K36M mutant cells overlaps with gene set of Regulation of apoptotic process, GO:0042981. The gene expressions in the two replicates of each cell line were firstly merged. Two independent clones of H3.3K36M and H3.1K36M were further merged for the analysis. The comparison was carried out among wild-type, H3.3K36M and H3.1K36M mutant cells, respectively.

(C) Gene expression levels in wild-type, H3.3K36M and H3.1K36M mutant cells. Red dots, the up-regulated genes with over 2-fold increased expression levels and p value less than 0.05. Blue dots, the down-regulated genes with over 2-fold decreased expression levels and p value less than 0.05.

**Figure S3. H3K36 methylations are reprogrammed in H3K36M mutant cells.**

(A) IGV views of H3K36me2 and H3K36me3 at BMP2, SOX5 and SXO9.

(B and C) Heatmaps illustrating H3K36me2 and H3K36me3 levels at H3K36me2 and H3K36me3 peaks in WT, H3.3K36M and H3.1K36M cells, respectively. H3K36me2 (B) and H3K36me3 (C) levels from 5 kb upstream to 5 kb downstream of the peak regions (in rows) were shown on a per-peak basis (in columns).

(D and E) The correlations of H3K36me2 (D) and H3K36me3 (E) among WT, H3.3K36M and H3.1K36M mutant cells. A 1kb sliding window spanning the whole genome was used to analyze the correlations of H3K36 methylations between different cell lines. The correlations were assessed by Pearson product moment correlation.

(F and G) The log2 fold change of gene expression was plotted against the occupancy alternations in H3K36me2 (F) and H3K36me3 (G) in H3.1K36M mutant cells. The correlations between gene expression and H3K36 methylation in H3.3K36M mutant cells was analyzed before in Fang *et al*., Science, 2016. Each dot indicated a single gene. R, correlation coefficient. The correlations were assessed by Pearson product moment correlation.

**Figure S4. H3K27me3 is re-located to new loci in H3.3K36M mutant cells.**

(A) The normalized reads density of H3K27me3 at intergenic regions. *** p < 0.001.

(B) The normalized reads density of H3K27me3 from 5 kb upstream to 5 kb downstream of gene bodies.

(C) Venn diagram illustration representing the overlap of H3K27me3 peaks in wild type, H3.3K36M and H3.1K36M mutant cells.

(D) The correlations of H3K27me3 among wild-type, H3.3K36M and H3.1K36M mutant cells. A 1 kb sliding window spanning the whole genome was used to calculate the correlations and R value is calculated as Pearson product moment correlation.

**Figure S5. H3K36M mutant proteins change the enhancers.**

(A) IGV views of H3K27me3, H3K27ac, H3K4me1 and H3K4me3 in wild-type and mutant cells.

(B) The distribution profiles of normalized H3K4me1 read density from 5 kb upstream of the TSS to 5 kb downstream of the TES. PRKM, reads per kilobase million.

(C) The normalized read densities of H3K4me1 at intergenic regions.

(D) Western blot of the whole cell protein in wild-type cells, H3.3K36M and H3.1K36M mutant cells.

(E) The lengths of enhancers in the wild-type cells, H3.3K36M and H3.1K36M mutant cells. bp, base pair. ***p < 0.001.

(F) The length distributions of the distances of common and unique enhancers to the closest TSS in H3.3K36M and H3.1K36M mutant cells. ***p < 0.001.

(G and H) The H3K27ac (G) and H3K4me1 (H) enrichments at active enhancers were analyzed via ChIP-PCR. The data are represented by the mean ± SD (N = 3 independent replicates). The analyzed loci were all identified as enhancer but with unique enrichments of H3K27ac in tested cell lines. WTE1, WTE2, and WTE3, enhancers with H3K27ac unique in wild-type cells. H3.3E1, H3.3E2, and H3.3E3, enhancers with H3K27ac unique in H3.3K36M-mutant cells. H3.1E1, H3.1E2, and H3.1E3, enhancers with H3K27ac unique in H3.1K36M-mutant cells. Intergenic, intergenic locus with low H3K27ac or H3K4me1 enrichment as a negative control.

(I and J) Correlations between the changes in H3K27ac and H3K27me3 at the increased H3K27me3 peaks in intergenic regions in H3.3K36M (I) and H3.1K36M (J) mutant cells.

(K) IGV views of H3K27me3, H3K27ac, H3K4me1 and H3K4me3 in wild-type and mutant cells at three unique primed enhancers in mutant cell, three unique poised enhancers in mutant cells. And three unique active enhancers in wild-type cells.

(L) Enrichments of H3K36M at active enhancers in wild-type cells that were within the H3K36me2 peaks and showed decreased H3K27ac in both H3.3K36M and H3.1K36M mutant cells. log2 (H3K27ac in mutant cells / H3K27ac in wild-type cells = -0.5 was chosen as the cutoff for the decrease of H3K27ac at active enhancers.

(M) Box plots representing the gene expression levels and the number of associated active enhancers.

(N) Enriched motifs in the inactivated enhancers in H3.3K36M and H3.1K36M mutant cells.

**Figure S6. H3.3K36M and H3.1K36M are incorporated into distinct chromatic loci.**

(A) Heatmaps illustrating H3K36M levels at H3.3K36M peak centers in H3.3K36M mutant cells and H3.1K36M peak centers in H3.1K36M mutant cells. H3K36M levels in 10 kb surrounding peak centers (in rows) were shown on a per-peak basis (in columns). H3K36M peaks in independent clones of H3.3K36M and H3.1K36M mutant were combined.

(B and C) The enrichments of H3K36M ChIP-seq at H3K36M peak centers in H3.3K36M (B) and H3.1K36M (C) cells. A 100bp sliding window was used to scan 10kb regions surrounding the peak center. ChIP-seq results in independent clones of H3.3K36M and H3.1K36M mutant were combined.

(D) The correlations of H3K36M among WT, H3.3K36M and H3.1K36M mutant cells. The correlations were assessed by Pearson product moment correlation.

**Figure S7. The changes of epigenome are determined by the genomic loci of H3K36M mutant proteins.**

(A) The normalized read densities of H3K36me3 at common and unique H3K36me2 peaks in H3.3K36M-mutant cells. A set of shuffled peaks corresponding to the unique peaks was used to analyze whether the enrichment of the ChIP-seq signals was significant compared to background enrichment. ***p < 0.001.

(B and C) The normalized read densities of H3.3K36M (B) and H3.1K36M (C) at common and unique H3K36me3 peaks in H3.3K36M-mutant cells. A set of shuffled peaks corresponding to the unique peaks was used to analyze whether the enrichment of the ChIP-seq signals was significant compared to background enrichment. ***p < 0.001.

(D - F) Same as (A) - (C), except the common and unique H3K36me2 peaks in H3.1K36M-mutant cells were analyzed. ***p < 0.001.

(G) The normalized reads density of H3K36me2 at H3K36me2 peaks common between WT and mutant cells. ChIP-seq results in independent clones of H3.3K36M and H3.1K36M mutant were combined. ***p < 0.001.

(H) The normalized reads density of H3K36me3 at H3K36me3 peaks common between WT and mutant cells. ChIP-seq results in independent clones of H3.3K36M and H3.1K36M mutant were combined. ***p < 0.001.

(I and J) RT-PCR analysis of the efficiencies of MMSET (I) and ASH1L (L) depletion. Two independent shRNAs were used to knock-down MMSET and ASH1L in wild-type, H3.3K36M and H3.1K36M mutant cells, respectively. ***p < 0.001.

(K) Correlations of changes of H3K27ac in H3.1K36M mutant cells and H3.3K36M at active enhancers in wild-type cells that were within the H3K36me2 peaks. The correlations were assessed by Pearson product moment correlation.

(L) Correlations of changes of H3K27ac in H3.3K36M mutant cells and H3.1K36M at active enhancers in wild-type cells that were within the H3K36me2 peaks. The correlations were assessed by Pearson product moment correlation.

(M) The schematic diagram of the impact of H3K36M on H3K36 methylation. The common H3K36 methylation peaks in wild-type and mutant cells were highly enriched with H3K36 methylation and the incorporation of H3K36M decreased the H3K36 methylation. The unique H3K36 methylation peaks in mutant cells were enriched with low amount of H3K36M mutant proteins which had mild impact on the methylation.

**Figure S8. The expression levels of dCas9-Trun H3K36M mutant protein were higher than H3.3K36M,R129E mutant.**

(A) The expression of low levels of H3.3K36M,R129E decreased H3K36me3/me2 in cells. Cell extracts were analyzed by Western blotting using the indicated antibodies.

(B) The expression level of dCas9-Trun H3K36M mutant protein was higher than ten times of H3.3K36M,R129E. Cell extracts were analyzed by Western blotting using the indicated antibodies. Different amount of H3.3K36M,R129E was tested. The 100 times loading didn’t show a clear band because the loaded protein was too much to separate the protein well. We didn’t compare this with the over-expression of dCas9-trun H3K36M proteins. *, Non-specific signal of Flag antibody.
