# Supplementary material for: The incorporation loci of H3.3K36M determine its preferential prevalence in chondroblastomas
Source: Cell Death Dis. 2021 Mar 24;12(4):311. doi: 10.1038/s41419-021-03597-9 (PMC7991640; doi:10.1038/s41419-021-03597-9)
Supplement: Supplementary file 11 — Supplementary table [file 41419_2021_3597_MOESM11_ESM.docx]

**Table S1. Primers used for qPCR.**

| **Primers** |  | **5' to 3'** | **Target** |
| --- | --- | --- | --- |
| BMP2 | Forward | ACCCGCTGTCTTCTAGCGT | BMP2 gene expression |
|  | Reverse | TTTCAGGCCGAACATGCTGAG |  |
| SOX9 | Forward | AGCGAACGCACATCAAGAC | SOX9 gene expression |
|  | Reverse | CTGTAGGCGATCTGTTGGGG |  |
| SOX5 | Forward | CAGCCAGAGTTAGCACAATAGG | SOX5 gene expression |
|  | Reverse | CTGTTGTTCCCGTCGGAGTT |  |
| RUNX2 | Forward | TGGTTACTGTCATGGCGGGTA | RUNX2 gene expression |
|  | Reverse | TCTCAGATCGTTGAACCTTGCTA |  |
| SMAD1 | Forward | AGAGACTTCTTGGGTGGAAACA | SMAD1 gene expression |
|  | Reverse | ATGGTGACACAGTTACTCGGT |  |
| WNT5A | Forward | ATTCTTGGTGGTCGCTAGGTA | WNT5A gene expression |
|  | Reverse | CGCCTTCTCCGATGTACTGC |  |
| ACAN | Forward | ACTCTGGGTTTTCGTGACTCT | ACAN gene expression |
|  | Reverse | ACACTCAGCGAGTTGTCATGG |  |
| COL2A1 | Forward | TGGACGATCAGGCGAAACC | COL2A1 gene expression |
|  | Reverse | GCTGCGGATGCTCTCAATCT |  |
| GAPDH | Forward | GGAGCGAGATCCCTCCAAAAT | GAPDH gene expression |
|  | Reverse | GGCTGTTGTCATACTTCTCATGG |  |
| BMP2 ChIP | Forward | CCACTGAGAAGAGTCCAGGTTC | BMP2 gene |
|  | Reverse | GGGGAGTATCAGGCCAATAGAG |  |
| SOX5 ChIP | Forward | TGTCAGTTTTCACCGTGCCT | SOX5 gene |
|  | Reverse | TTTCCGTCCCCTCAACAGTG |  |
| SOX9 ChIP | Forward | TTCTTCTTCCTTAAAGACATTTAAG | SOX9 gene |
|  | Reverse | GGATAGGTCATGTTTGTGTCTTGG |  |
| RUNX2 ChIP | Forward | CTCAGCCAGCCCATCTACAG | RUNX2 gene |
|  | Reverse | AACACAGTGGCCTTGGAGG |  |
| SMAD1 ChIP | Forward | CATGCTGCTAACCACTGTGC | SMAD1 gene |
|  | Reverse | TCATGCAGCTGGTGCACATA |  |
| IREB2 ChIP | Forward | GCGATTTCCAGGCTTGCTTAAA | IREB2 gene |
|  | Reverse | AAAGCGGGTTGAAAAACACGG |  |
| ACR ChIP | Forward | TCTCACTGCTGCTTGGATGG | ACR gene |
|  | Reverse | GACAGAAGGGTCCCCAACAG |  |
| ZSWIM6 ChIP | Forward | AGGAAGGAACGCAGTGTGAG | ZSWIM6 gene |
|  | Reverse | CACTCTTGCCCTCACCAACA |  |
| RCL1 ChIP | Forward | AAGGGTCATGCCAGAGCAAG | RCL1 gene |
|  | Reverse | GCAGAGCATCCAAAAGCAGG |  |
| NFIB ChIP | Forward | AACTCACATGGGTGGGGAAGT | NFIB gene |
|  | Reverse | CCATTTAGACAATGATAGGTGGC |  |
| JAK2 ChIP | Forward | GCTTGTGCAAAGAGGTGAGC | JAK2 gene |
|  | Reverse | GCTGGTTTCTGCCTCCATCT |  |
| PTPRD ChIP | Forward | ACGTCAGAGCAAACAGCAGA | PTPRD gene |
|  | Reverse | GTGTCCCCAGTCTTACTGCC |  |
| TET1 ChIP | Forward | AGACAGCATGACAGGCATCG | TET1 gene |
|  | Reverse | AGCAACAGAGCTCATAGCGT |  |
| ASAP2 ChIP | Forward | TGAATCACGGAACTAGCAAACT | ASAP2 gene |
|  | Reverse | GTACAGGATAAGGGCCCAACAG |  |
| CLCA1 ChIP | Forward | TCGATTGCAACGACAATGCC | CLCA1 gene |
|  | Reverse | TCGATTGCAACGACAATGCC |  |
| WTE1 ChIP | Forward | TATTAAGCACCAGCCACGGG | chr1:56,179,928-56,187,864 |
|  | Reverse | AGACTCGGGTCCATCAGACA |  |
| WTE2 ChIP | Forward | AGTGGGAAGGATGGCCCTAT | chr9:127,258,692-127,276,680 |
|  | Reverse | GAGTACCCGGAGCCTTATGC |  |
| WTE3 ChIP | Forward | AGCCCTGTCCCTCCTGATAG | chr13:75,326,508-75,382,541 |
|  | Reverse | CAGCGTGGATTGAGGGAGTT |  |
| H3.3E1 ChIP | Forward | TTACCGCCTCACCAAAGCAA | chr5:162,688,721-162,710,725 |
|  | Reverse | GTGCACCAACACAACTTCGG |  |
| H3.3E2 ChIP | Forward | TGCAGCCATGTATGGGTTGT | chr6:93,661,340-93,671,745 |
|  | Reverse | GTGCCGGGCTATTACCACTT |  |
| H3.3E3 ChIP | Forward | GAGTGACGGCAACTGTGGTA | chr7:73,437,330-73,447,465 |
|  | Reverse | TGGCTCCGAGGGATGAGTTA |  |
| H3.1E1 ChIP | Forward | GTTGGCCACAGTGACAAAATCC | chr5:55,687,683-55,693,183 |
|  | Reverse | GACTCTTCAGGGAAGACACG |  |
| H3.1E2 ChIP | Forward | TTGGTACAGGCTCCAGAGGT | chr5:157,992,962-158,003,963 |
|  | Reverse | CGTCTGGATGTTTGGCAACG |  |
| H3.1E3 ChIP | Forward | GCTGCCGACAACATTTCCAA | chr6:94,994,465-94,999,667 |
|  | Reverse | GTGTCTTGTCCCATCCCGAC |  |
| Intergenic ChIP | Forward | GGAGGTGGTGTACAGTCATGC | chr13:82,875,968-82,882,971 |
|  | Reverse | GGGTTCCTGACATGGCTTTCTA |  |
| Ac1 ChIP | Forward | AAGACACTGAAGAGCCTCGC | chr19:28,914,333-28,923,086 |
|  | Reverse | AACAGTAGCTGAGTTCCGGC |  |
| Ac2 ChIP | Forward | GTCCTCAGGTCAAGGTCACG | chr21:46,730,804-46,745,054 |
|  | Reverse | GCACCACACAGAGATCCGAA |  |
| Ac3 ChIP | Forward | GCGATACTGACTCCCCATCA | chr6:141,174,777-141,187,540 |
|  | Reverse | TGCCTTCTTAGCCCACGTTC |  |
| Pr1 ChIP | Forward | GAGACTGCATGGGCCTCTTT | chr6:141,164,793-141,171,174 |
|  | Reverse | ATGCACTGGGGAAACGTAGG |  |
| Pr2 ChIP | Forward | TAGGGGAAGAGCAAACCCCT | chr3:70,878,912-70,886,296 |
|  | Reverse | CAGGCTTTCCCGGGATCTAC |  |
| Pr3 ChIP | Forward | GGAGGCTCCTTACTCTACTCCA | chr2:108,814,436-108,818,132 |
|  | Reverse | TTCTCAGTTATCCAGCGGGTAG |  |
| Po1 ChIP | Forward | CAAAGTCGCCAAGTTCCAGC | chr21:34,485,596-34,499,846 |
|  | Reverse | CATCCGAACTCTTGTGGGCT |  |
| Po2 ChIP | Forward | TCACGCGGATGTTAAGTCCC | chr6:126,079,479-126,105,006 |
|  | Reverse | GAGAAACGCTCTCAGCTGGT |  |
| Po3 ChIP | Forward | CATTTGGTAGAACAACAGGCAGA | chr3:132,106,246-132,113,630 |
|  | Reverse | GGGAGAATGCTGAGAATGTTGTAT |  |
| NE ChIP | Forward | GGAGGCTCCTTACTCTACTCCA | chr2:108,814,436-108,818,132 |
|  | Reverse | TTCTCAGTTATCCAGCGGGTAG |  |
| IREB2-target | Forward | TTCTGCCTTACTCAATACGGGT | dCas9 targeting test |
|  | Reverse | ATCTTGAAGAAGAACACGGGCA |  |
| BMP2-target | Forward | GTCCTGAGCGAGTTCGAGTT | dCas9 targeting test |
|  | Reverse | GACCTGAGTGCCTGCGATAC |  |
| IREB2-sg1 | Forward | AACAAGACCCGTATTGAGTA | sgRNA for IREB2 |
| IREB2-sg2 | Forward | GCTGCTGTACGAAATTGTGA | sgRNA for IREB2 |
| IREB2-sg3 | Forward | ATGTTCTGCCTTACTCAATA | sgRNA for IREB2 |
| BMP2-sg1 | Forward | GCCGCAACTCGAACTCGCTC | sgRNA for BMP2 |
| BMP2-sg2 | Forward | ACGCCGCCGCGAACTTCCTG | sgRNA for BMP2 |
| BMP2-sg3 | Forward | TCCTGAGCGAGTTCGAGTTG | sgRNA for BMP2 |
| BMP2-sg4 | Forward | ACATGCTAGACCTGTATCGC | sgRNA for BMP2 |
| IREB2-RT | Forward | TCGATGTATCTAAACTTGGCACC | IREB2 gene expression |
|  | Reverse | GCCATCACAATTTCGTACAGCAG |  |

**Table S2. Correlations between two replicates of ChIP-seq in each cell line. A 1kb sliding window across the whole genome was used to calculated the Pearson product moment correlation.**

|  | T/C28a2 | H3.3K36M #1 | H3.3K36M #2 | H3.1K36M #1 | H3.1K36M #2 |
| --- | --- | --- | --- | --- | --- |
| H3K36me2 | 0.81 | 0.82 | 0.77 | 0.74 | 0.84 |
| H3K36me3 | 0.88 | 0.7 | 0.85 | 0.88 | 0.86 |
| H3K36M | 0.76 | 0.81 | 0.77 | 0.82 | 0.79 |
| H3K27me3 | 0.72 | 0.79 | 0.75 | 0.79 | 0.73 |
| H3K27ac | 0.81 | 0.83 | 0.76 | 0.74 | 0.82 |
| H3K4me1 | 0.83 | 0.85 | 0.78 | 0.76 | 0.81 |
| H3K4me3 | 0.78 | 0.79 | 0.77 | 0.81 | 0.82 |
